# Supplementary material for: DHODH inhibition modulates glucose metabolism and circulating GDF15, and improves metabolic balance
Source: iScience. 2021 May 1;24(5):102494. doi: 10.1016/j.isci.2021.102494 (PMC8169992; doi:10.1016/j.isci.2021.102494)
Supplement: Document S1. Figures S1–S6 [file mmc1.pdf]

iScience, Volume *24*

Supplemental information

DHODH inhibition modulates glucose metabolism and circulating GDF15, and improves metabolic balance

Juan Zhang, Graciela Terán, Mihaela Popa, Harsha Madapura, Marcus James Graeme Watson Ladds, Danai Lianoudaki, Jacob Grünler, Marie Arsenian-Henriksson, Emmet McCormack, Martin Enrique Rottenberg, Sergiu-Bogdan Catrina, Sonia Laín, and Suhas Darekar

SUPPLEMENTAL FIGURES

A

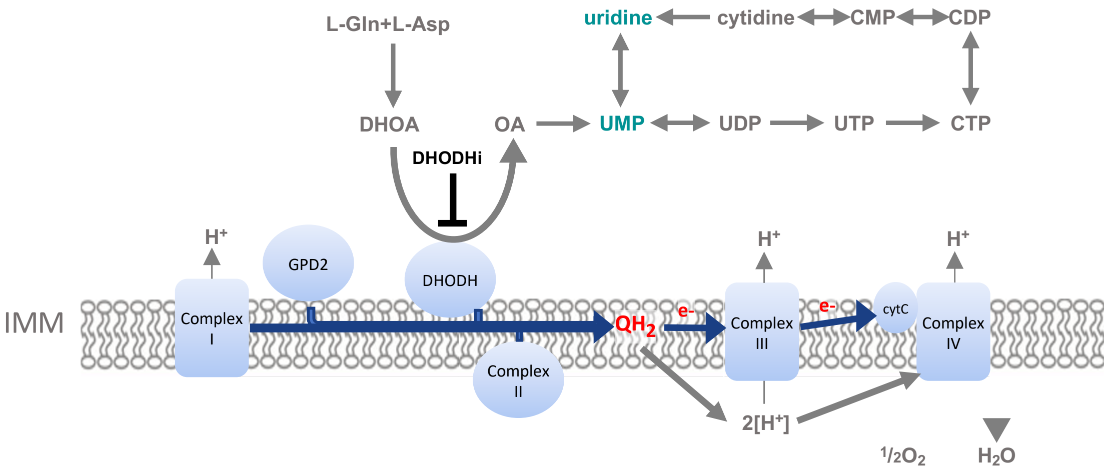

B

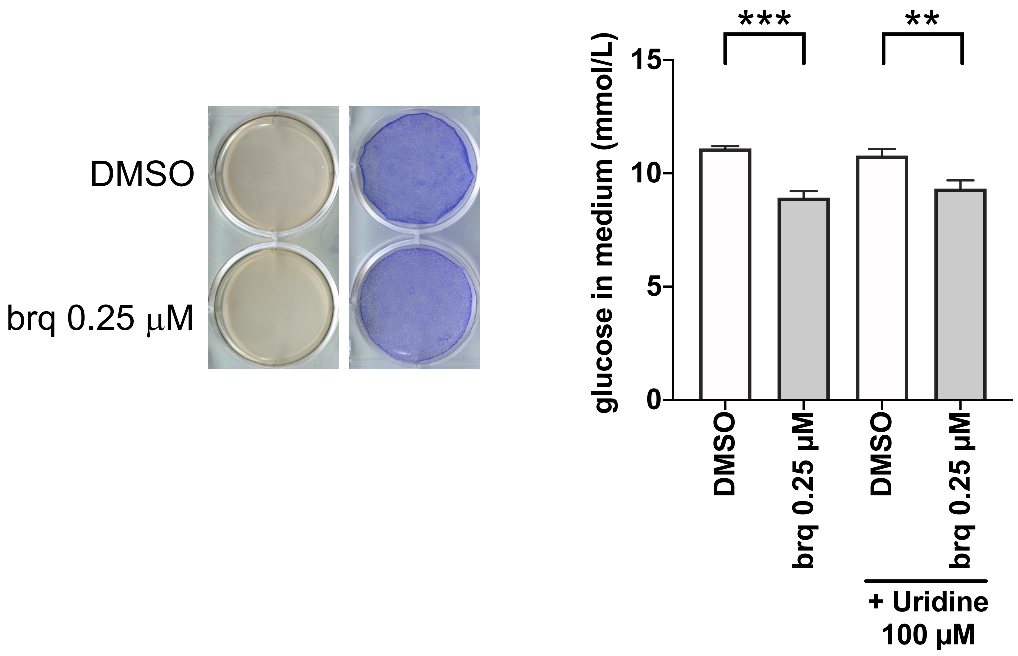

Figure S1. (A) Scheme depicting the function of DHODH on pyrimidine ribonucleotide synthesis and on the mitochondrial respiratory chain. IMM= inner mitochondrial membrane. Related to Figure 1.
(B) Brequinar reduces glucose concentration in the cell culture medium. Murine T22-RGCΔFos-LacZ fibroblasts were grown in the presence of brequinar. On day 3 after treatment, pictures of the plates were taken and Giemsa stainings were performed to assess cell density. Concentration of glucose in the medium was determined. Values correspond to the mean of three technical repeats. p values were calculated by Student's t test and error bars indicate SD. Related to Figure 1.

Figure S2

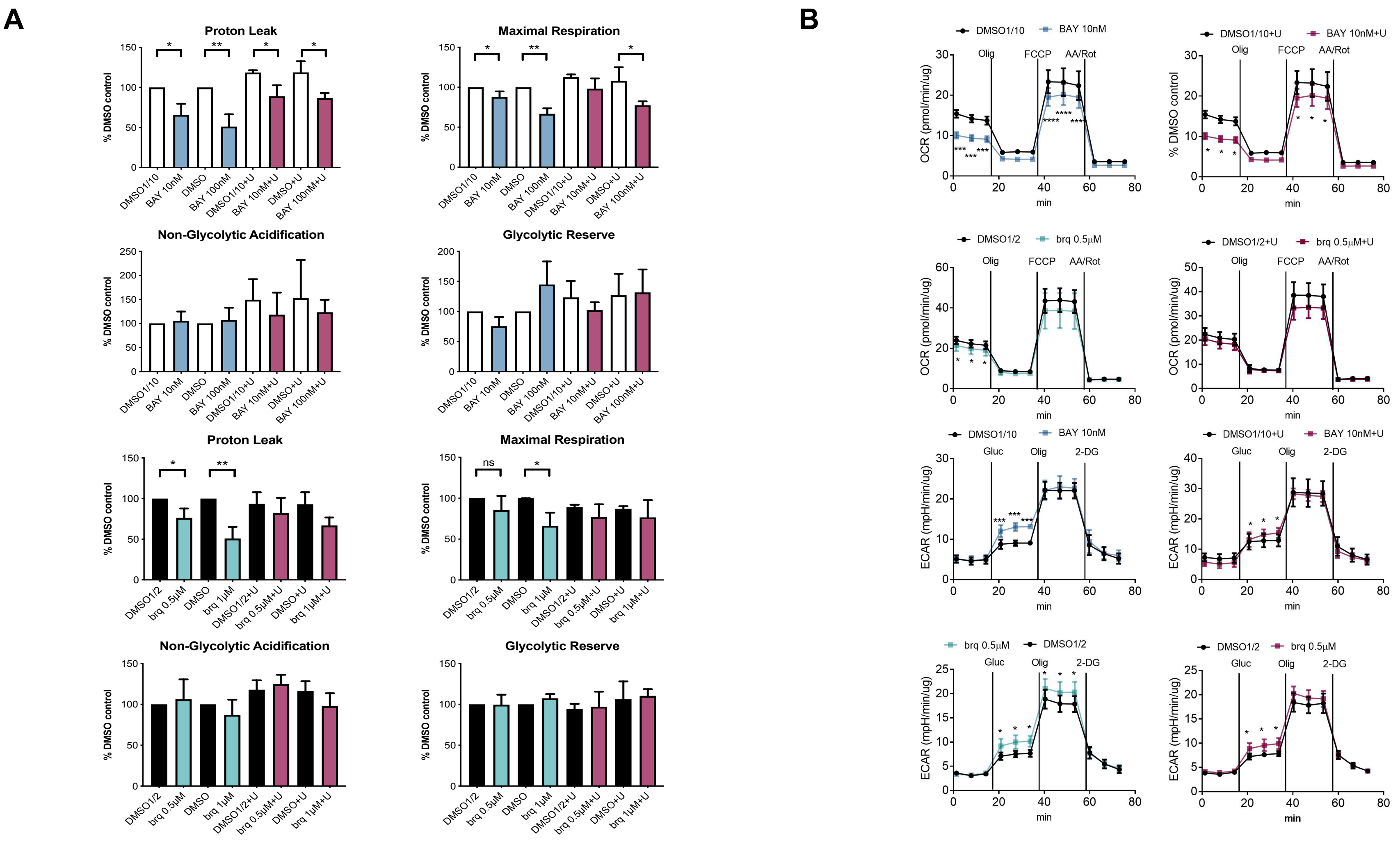

Figure S2. Cellular respiration and glycolysis measurements related to Figure 1C.

(A) Cellular respiration and glycolysis measurements. Values correspond to the average of 3 biological repeats and are relative to the corresponding DMSO vehicle control. Error bars represent SD and p values correspond to Student's t test. Related to Figure 1B.

(B) Average (±SEM) oxygen consumption rate (OCR) and extracellular acidification rate (ECAR) measurements. n=3 biological repeats. +U, +100 μM uridine. Related to Figure 1B.

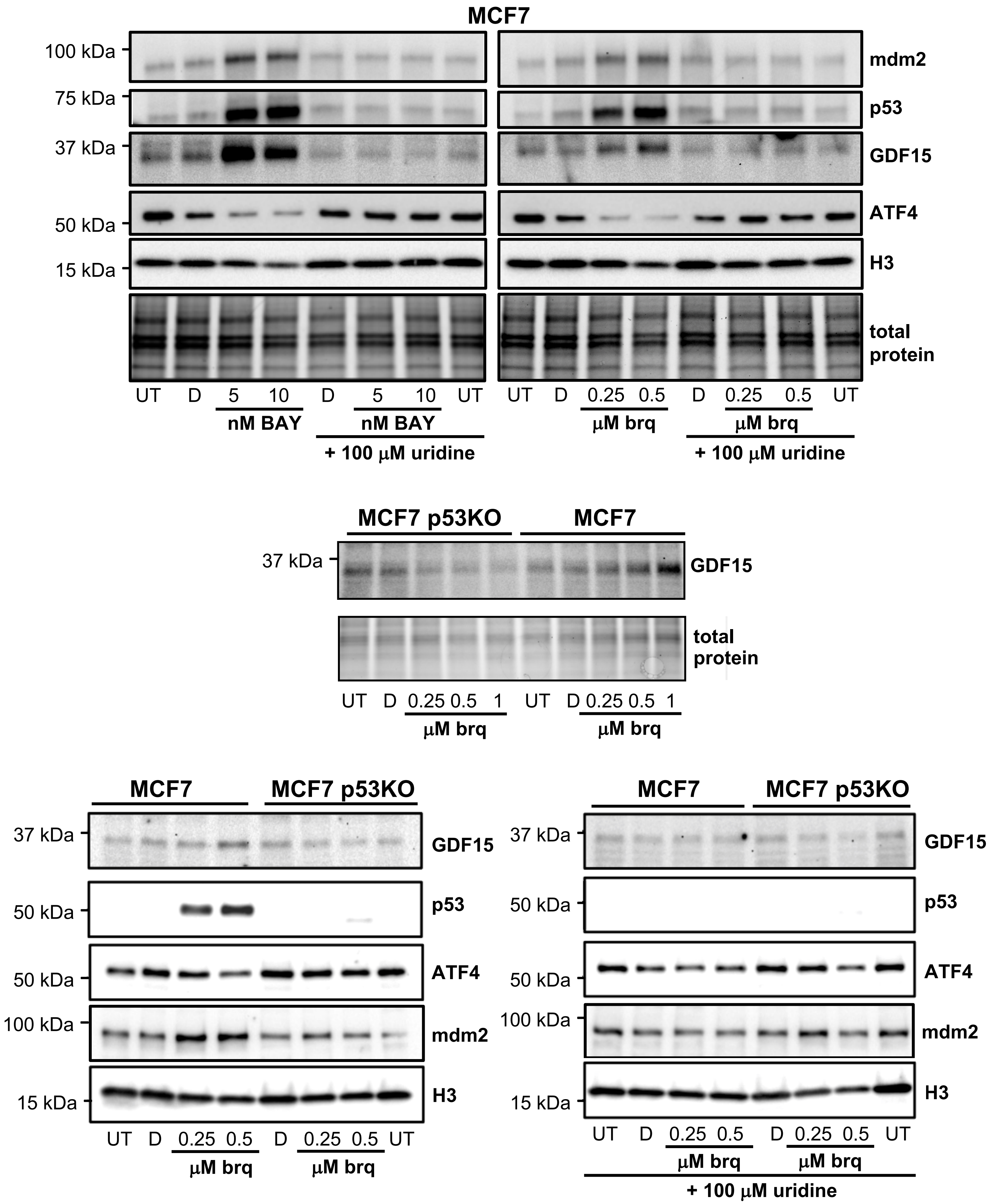

Figure S3. MCF7 or MCF7 p53KO cells were treated as indicated for 48 h. Expression of mdm2, p53, ATF4 and GDF15 were detected by western blotting. Total protein and histone H3 were used as loading controls. UT, untreated; D, DMSO. Related to Figure 2A.

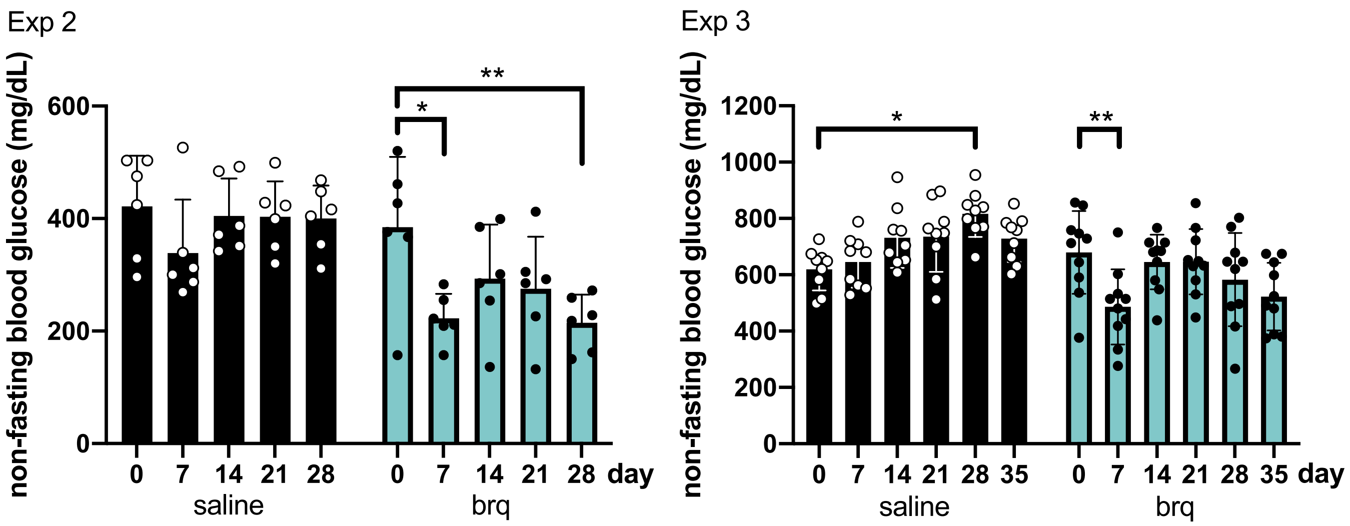

Figure S4. Effect of DHODH inhibitors on non-fasting blood glucose. Average (±SD) non-fasting blood glucose levels in experiments 2 and 3. Only significant changes are indicated. p values are calculated using 2way Anova. Note that in the brequinar experiments (this inhibitor is administered every three days) significance is achieved only on the days after injection (days 7 and 28). Related to Figure 5.

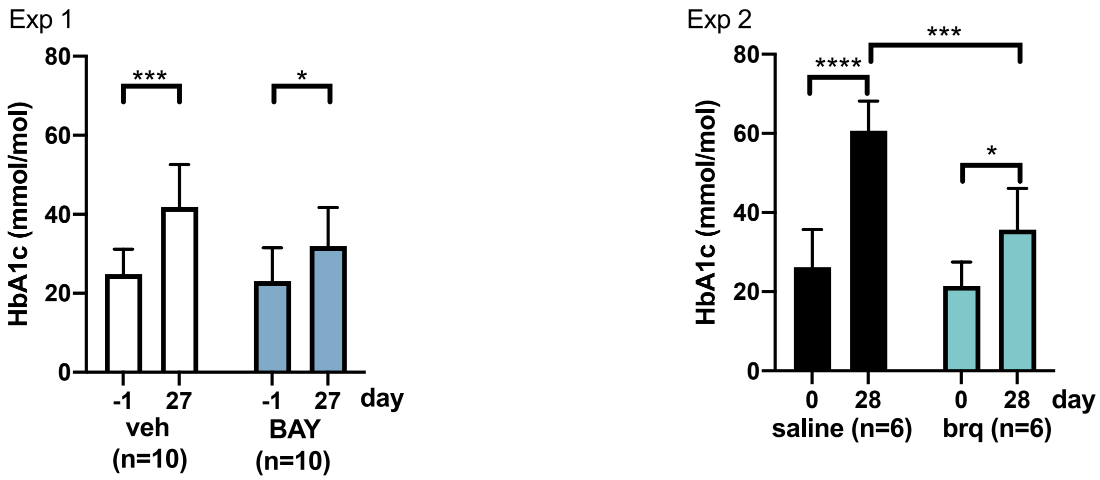

Figure S5. Additional data for Figure 5B. Average (±SD) HbA1c levels at the indicated days after treatment in experiments 1 and 2. p values by Student's t test. Day 0 is the treatment start day. Related to Figure 5B.

Figure S6

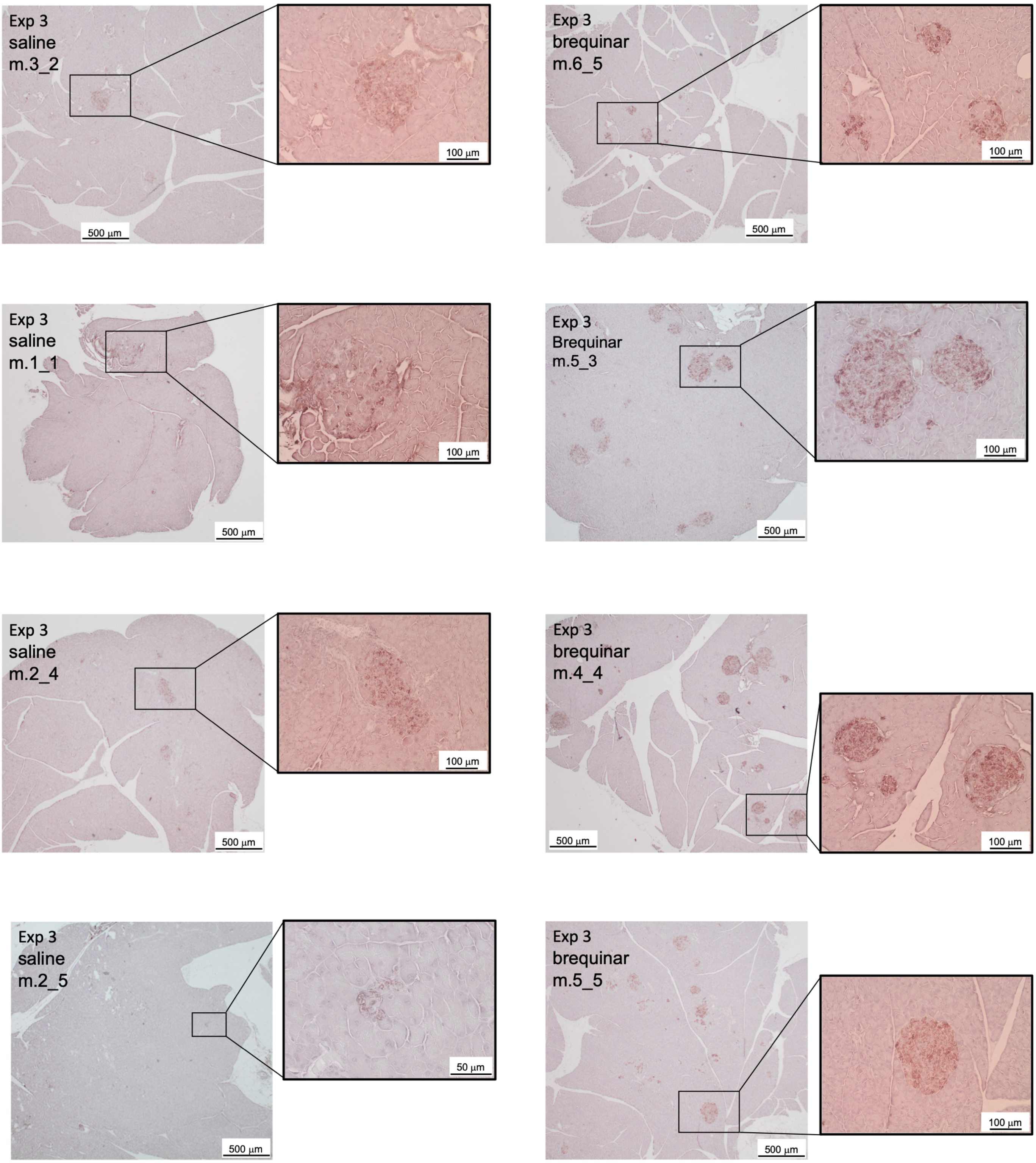

Figure S6. Representative staining of pancreases from different mice in experiment 3 with an antibody against insulin. Related to Figures 6B and 6C.
