# Supplementary material for: DHODH inhibition modulates glucose metabolism and circulating GDF15, and improves metabolic balance
Source: iScience. 2021 May 1;24(5):102494. doi: 10.1016/j.isci.2021.102494 (PMC8169992; doi:10.1016/j.isci.2021.102494)
Supplement: Document S2. Article plus supplemental information [file mmc2.pdf]

iScience

CellPress
OPEN ACCESS

Article

# DHODH inhibition modulates glucose metabolism and circulating GDF15, and improves metabolic balance

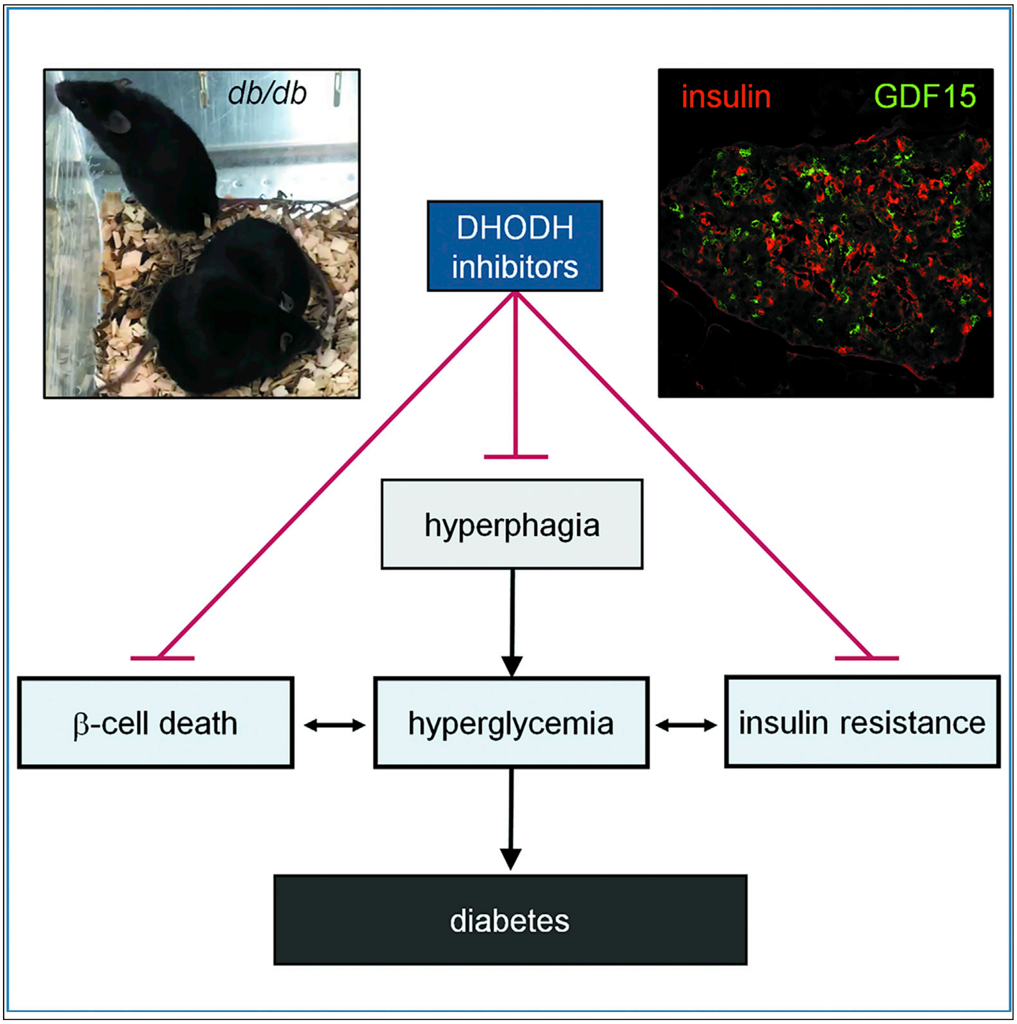


Juan Zhang, Graciela Terán, Mihaela Popa, ..., Sergiu-Bogdan Catrina, Sonia Laín, Suhas Darekar

suhas.darekar@ki.se

## Highlights

DHODH inhibitors impair respiration and increase glycolysis.

They promote the expression and secretion of GDF15 in a p53-dependent manner.

In hyperphagic *db/db* mice, DHODH inhibitors increase circulating GDF15.

They protect pancreatic β cells and improve glucose balance in these mice.

Zhang et al., iScience *24*, 102494
May 21, 2021 © 2021 The Author(s).
https://doi.org/10.1016/j.isci.2021.102494

Check for updates

iScience

CellPress
OPEN ACCESS

Article

# DHODH inhibition modulates glucose metabolism and circulating GDF15, and improves metabolic balance

Juan Zhang,[1] Graciela Terán,[1] Mihaela Popa,[3] Harsha Madapura,[1,2] Marcus James Graeme Watson Ladds,[1] Danai Lianoudaki,[1,2] Jacob Grünler,[6,7] Marie Arsenian-Henriksson,[1] Emmet McCormack,[3,4] Martin Enrique Rottenberg,[1] Sergiu-Bogdan Catrina,[5,6,7] Sonia Laín,[1,2,8] and Suhas Darekar[1,2,8,9,*]

## SUMMARY

**Dihydroorotate dehydrogenase (DHODH) is essential for the *de novo* synthesis of pyrimidine ribonucleotides, and as such, its inhibitors have been long used to treat autoimmune diseases and are in clinical trials for cancer and viral infections. Interestingly, DHODH is located in the inner mitochondrial membrane and contributes to provide ubiquinol to the respiratory chain. Thus, DHODH provides the link between nucleotide metabolism and mitochondrial function. Here we show that pharmacological inhibition of DHODH reduces mitochondrial respiration, promotes glycolysis, and enhances GLUT4 translocation to the cytoplasmic membrane and that by activating tumor suppressor p53, increases the expression of GDF15, a cytokine that reduces appetite and prolongs lifespan. In addition, similar to the antidiabetic drug metformin, we observed that in *db/db* mice, DHODH inhibitors elevate levels of circulating GDF15 and reduce food intake. Further analysis using this model for obesity-induced diabetes revealed that DHODH inhibitors delay pancreatic β cell death and improve metabolic balance.**

## INTRODUCTION

Pharmacologic inhibition of DHODH with leflunomide has been used to treat patients with autoimmune diseases since the 1990s. In addition, leflunomide shows anticancer activity in animal models (Brown et al., 2017; Mathur et al., 2017; White et al., 2011). However, the use of leflunomide and teriflunomide (the active metabolite of leflunomide) is limited by off-target effects and a remarkably long half-life, which requires the use of either cholestyramine or charcoal to enhance clearance in the case of hypersensitivity (Alcorn et al., 2009; Brent, 2001; Doscas et al., 2014). Recent work with brequinar, a highly specific and potent DHODH inhibitor described in the 1980s, has led to the understanding that DHODH inhibitors could be effective in acute myeloid leukemia cases (Sykes et al., 2016). In this regard, there is a clinical trial ongoing using a novel and extremely potent DHODH inhibitor (BAY2402234 [Christian et al., 2019]) for myeloid malignancies (clinicaltrials.gov). This renewed interest in DHODH as a target for cancer has been accompanied by the discovery of numerous inhibitors in addition to the long list of agents already described (Boukalova et al., 2020; Ladds et al., 2018; Mollick and Laín, 2020; Munier-Lehmann et al., 2013). Moreover, inhibitors of DHODH, including leflunomide, are also considered to combat RNA virus infections (Deans et al., 2016; Xiong et al., 2020) and have now entered clinical trials for coronavirus disease 2019 (COVID-19) (clinicaltrials.gov).

DHODH catalyzes the oxidation of dihydroorotate to orotate, which is further converted into uridine monophosphate from which all other pyrimidine ribonucleotides can be obtained. Depletion of pyrimidine ribonucleotide pools is thought to be the main reason why DHODH inhibitors reduce RNA virus replication and slow down the proliferation of fast-growing cells such as activated T cells and cancer cells. Supporting this, the effects of DHODH inhibitors on viral load and cell proliferation can be abolished by the addition of an excess of uridine (50 μM−1 mM) through a salvage pathway (Figure S1A) (Christian et al., 2019; Deans et al., 2016; Ladds et al., 2018; Sykes et al., 2016). One special feature of DHODH is that in mammalian cells, this enzyme is found in the inner mitochondrial membrane (Figure S1A) and is the only enzyme in the pyrimidine ribonucleotide synthesis pathway at this site. The oxidation of dihydroorotate to orotate catalyzed by DHODH occurs by transferring electrons to ubiquinone (Rawls et al., 2000). In this way, DHODH provides reduced ubiquinone (ubiquinol) to complex III of the respiratory chain (Fang et al., 2013; Vélez et al., 2013).

[1]Department of Microbiology, Tumor and Cell Biology (MTC), Biomedicum, Karolinska Institutet, SE-171 65 Stockholm, Sweden

[2]SciLifeLab, Department of Microbiology, Tumor and Cell Biology (MTC), Karolinska Institutet, Tomtebodavägen 23, SE-171 21 Stockholm, Sweden

[3]Centre for Cancer Biomarkers, CCBIO, Department of Clinical Science, Hematology Section, University of Bergen, 5021 Bergen, Norway

[4]Department of Medicine, Haematology Section, Haukeland University Hospital, Bergen, Norway

[5]Department of Molecular Medicine and Surgery, Karolinska Institutet, 17176 Stockholm, Sweden

[6]Department of Endocrinology and Diabetes, Karolinska University Hospital, 17176 Stockholm, Sweden

[7]Center for Diabetes, Academic Specialist Centrum, 11365 Stockholm, Sweden

[8]These authors contributed equally

[9]Lead contact

*Correspondence: suhas.darekar@ki.se

https://doi.org/10.1016/j.isci.2021.102494


This is an open access article under the CC BY license (http://creativecommons.org/licenses/by/4.0/).

Given its ubiquinone dependency and localization, DHODH activity supports respiratory chain function (Gattermann et al., 2004; Khutornenko et al., 2010; Löffler et al., 1997) and also provides the link that balances respiration and pyrimidine ribonucleotide synthesis (Figure S1A).

According to the World Health Organization, the number of people with diabetes rose from 108 million in 1980 to 422 million in 2014 with the majority of them suffering from type 2 diabetes. Metformin is still the first-line therapeutic for type 2 diabetes, and although still in debate, its ability to weaken mitochondrial respiration has been considered to be important for its antidiabetic effect (Bridges et al., 2016; Madiraju et al., 2014; Owen et al., 2000; Wang et al., 2019). In addition, metformin can increase circulating growth/differentiation factor 15 (GDF15) levels (Gerstein et al., 2017). GDF15 is produced by a wide range of cell types and is associated with several pathophysiological conditions; however, rather than causing them, it is potentially involved in protecting tissues from further damage (Tsai et al., 2018). Interestingly, transgenic mice overexpressing GDF15 live longer, which suggests that overall, the effect of GDF15 can be beneficial (Wang et al., 2014). GDF15 binds to the GFRAL receptor (GFRAL) (Mullican et al., 2017) and activates GFRAL-expressing brainstem neurons ultimately leading to a reduction in food intake. Of relevance to the work described here, metformin reduces food intake and body weight in humans as well as in mice and does so in a manner that is dependent on its ability to increase circulating GDF15 (Coll et al., 2020; Day et al., 2019). In leptin receptor-deficient *db/db* mice, GDF15 depletion associates with renal damage leading to higher blood glucose, glucosuria, polyuria, and polydipsia (Mazagova et al., 2013). Altogether, these studies suggest that GDF15 protects from type 2 diabetes. In addition, in type 1 diabetes, GDF15 may enhance insulin production by protecting the pancreas from inflammation (Nakayasu et al., 2020).

Given that DHODH participates in mitochondrial respiration, that GDF15 expression is induced by the tumor suppressor p53 (Li et al., 2000), that DHODH inhibitors increase p53 synthesis (Ladds et al., 2018; Popova et al., 2020), and that an extra *TP53* allele can delay aging in mice (Matheu et al., 2007), here we tested the effects of DHODH inhibitors on metabolic balance and on the production of GDF15 by cells and in *db/db* mice as a model for obesity-induced type 2 diabetes.

## RESULTS

### DHODH inhibitors reduce oxygen consumption and increase glycolysis

We observed that when cells were cultured in the presence of DHODH inhibitor, the culture medium became acidified and that there was a reduction in the concentration of glucose in the medium (Figure S1B). This suggested an increase in lactate production and an increase in glucose consumption by cells. Accordingly, and as shown in Figure 1A, brequinar, like insulin and metformin, induced the translocation of the glucose transporter GLUT4 to the plasma membrane. Supporting that the effect of brequinar was due to inhibition of DHODH, BAY2402234 had the same effect on GLUT4. As induction of the translocation of GLUT4 to the plasma membrane is also a feature of the mitochondrial complex I inhibitor rotenone (Becker et al., 2001) and DHODH is involved in mitochondrial respiration, we measured oxygen consumption rate (OCR) and extracellular acidification rates in the cell culture medium and observed that both DHODH inhibitors (BAY2402234 and brequinar) partially reduced OCR and promoted a shift toward glycolysis (Figures 1B, 1C, and S2).

When cells were given a large excess of uridine (100 μM), which thwarts the effect of DHODH inhibitors on cell proliferation (Ladds et al., 2018), the effects of brequinar and BAY2402234 on respiration and glycolysis were not fully prevented (Figures 1B, 1C, and S2). As could be expected (see Figure S1A), this suggests that the disruption of mitochondrial respiratory function by DHODH inhibitors is less sensitive to uridine supplementation than their effect on cell proliferation. Another aspect that may be of relevance is that brequinar promotes mitochondrial fusion, a feature that could affect respiration efficacy (Miret-Casals et al., 2018).

### DHODH inhibitors increase GDF15 levels

Figures 2A and S3 show that BAY2402234 and brequinar elevate intracellular GDF15 levels in MCF7 human breast cancer cells. GDF15 was also increased by these two DHODH inhibitors in the medium of MCF7 cultures as well as in the medium of murine fibroblast cultures (Figures 2B and 2C). The increase in both intracellular and secreted GDF15 was ablated by an excess of uridine. This demonstrates that DHODH inhibitors increase the synthesis and/or secretion of GDF15 by blocking pyrimidine ribonucleotide synthesis.

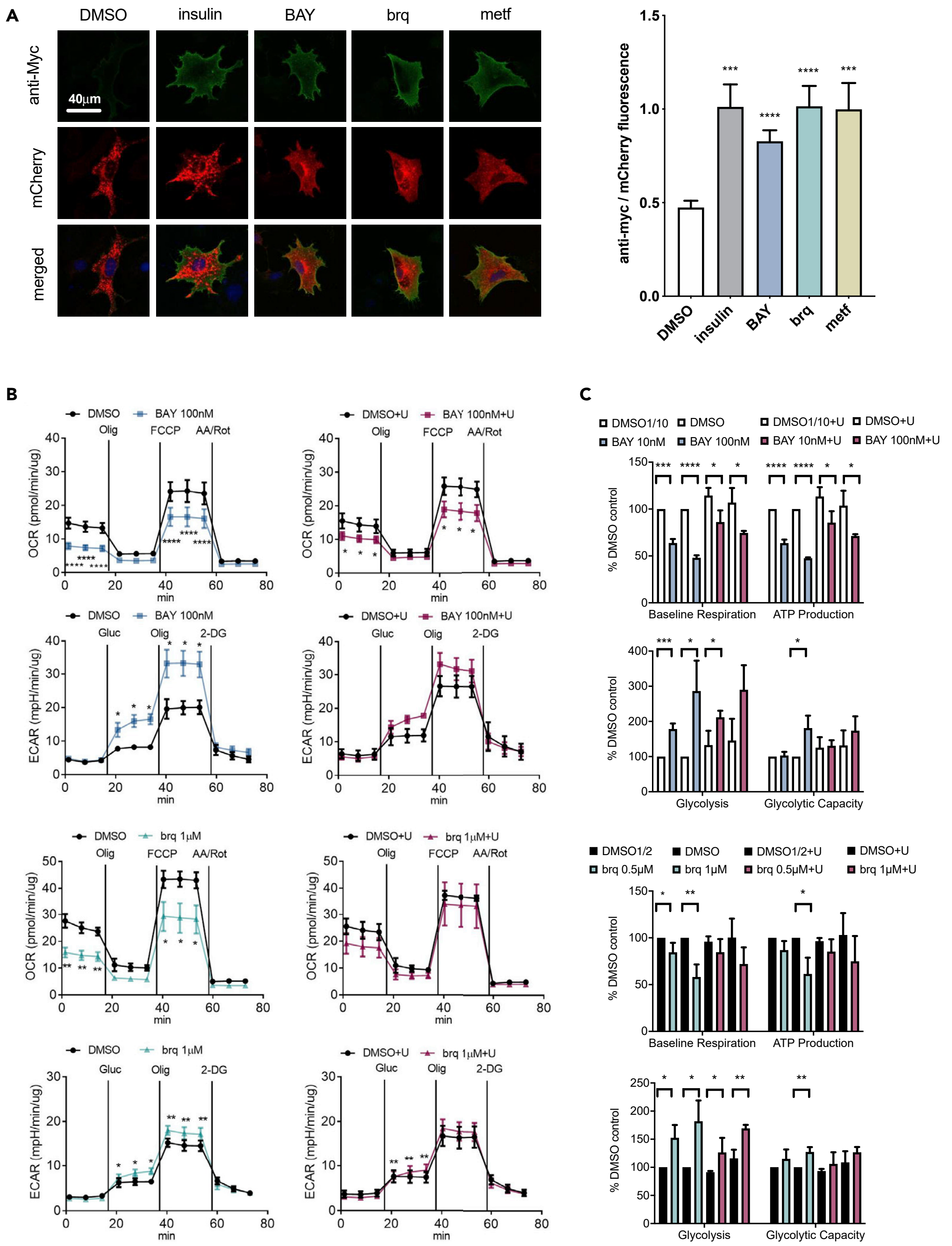


**Figure 1. DHODH inhibitors promote GLUT4 translocation to the plasma membrane and affect mitochondrial respiration and glycolysis**

(A) Localization of GLUT4 upon treatment with the indicated compounds. Plasma membrane-bound GLUT4 is labeled with a Myc tag on its extracellular domain, and total GLUT4 is labeled with mCherry. The average (±SEM) of the ratio between anti-Myc and mCherry fluorescence was calculated. p values correspond to Student's t test, and n = 23–30 cells for each treatment.

(B and C) Cellular respiration and glycolysis measurements.

(B) Average (±SEM) oxygen consumption rate (OCR) and extracellular acidification rate (ECAR) measurements.

(C) Variation of respiration and glycolysis parameters in response to the indicated inhibitors. Values correspond to the average (±SD). n = 3 biological repeats, and p values correspond to Student's t test. See also Figure S2. +U, +uridine 100 μM; BAY, BAY2402234; brq, brequinar

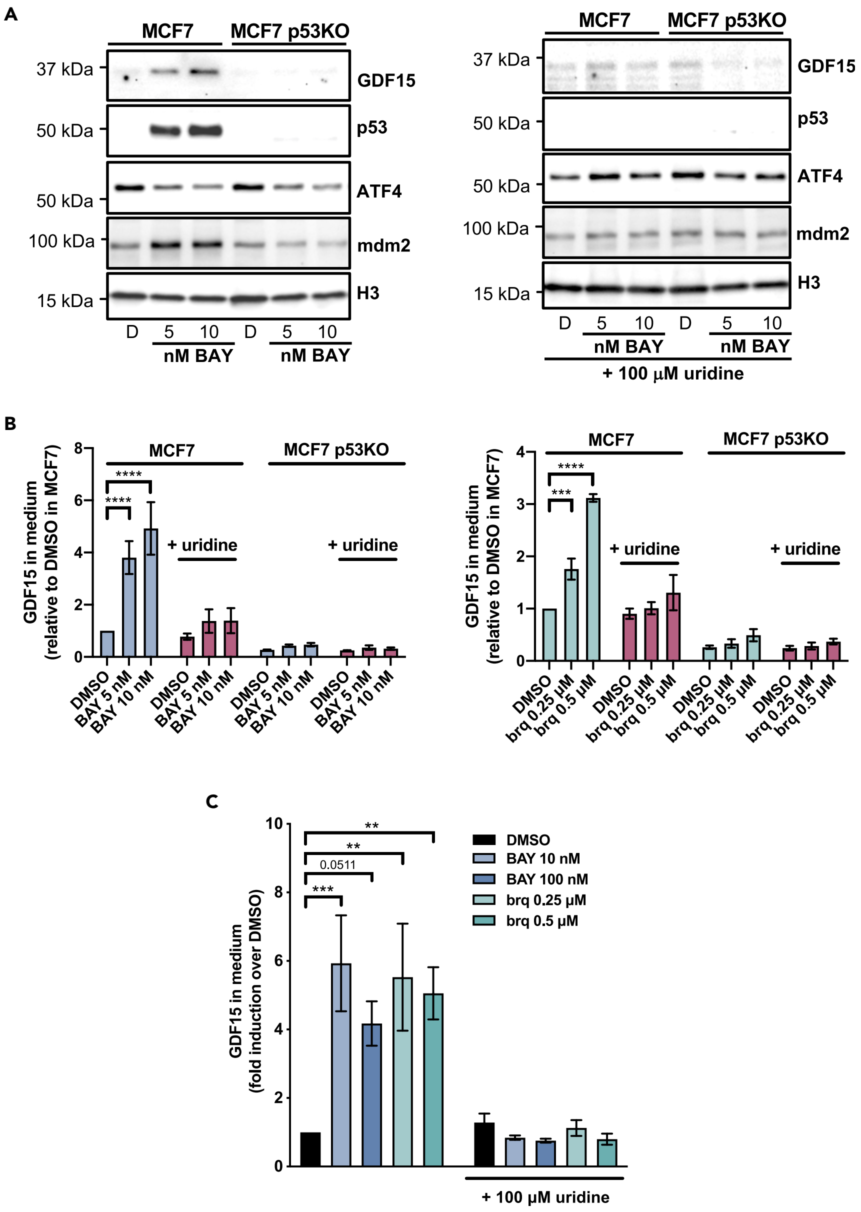


**Figure 2. DHODH inhibitors increase GDF15 expression and secretion**

(A) Expression of GDF15, p53, ATF4, and mdm2 were measured by western blotting of MCF7 and MCF7 p53KO cell extracts from cultures treated for 48 h as indicated. Histone H3 was used as loading control.

(B and C) MCF7 cells, MCF7 p53KO cells, or T22-RGCΔFos-LacZ murine fibroblasts were treated for 48 h as indicated, after which GDF15 in the culture medium was measured. Where indicated 100 µM uridine was added. Error bars correspond to SEM of four biological repeats, and p values were calculated by two-way ANOVA.

See also Figure S3.

As mentioned earlier, transcription of GDF15 is known to be induced by p53 (Li et al., 2000), DHODH inhibitors activate p53, and this is prevented in the presence of 100 µM uridine (Ladds et al., 2018; Popova et al., 2020). In line with these observations and as shown in Figures 2A, 2B, and S3, both DHODH inhibitors increased intracellular and secreted GDF15 levels in wild-type p53-expressing MCF7 cultures but not in MCF7 p53 knockout cells. ATF4 is another transcription factor activating GDF15 expression (Patel et al., 2019), but we did not detect an increase in its levels in response to DHODH inhibitors (Figures 2A and S3). However, it is interesting to note that mdm2, whose expression is activated by p53 and DHODH inhibitors (Ladds et al., 2018) (see Figures 2A and S3), binds to and drives the transcription factor activity of ATF4 (Riscal et al., 2016).

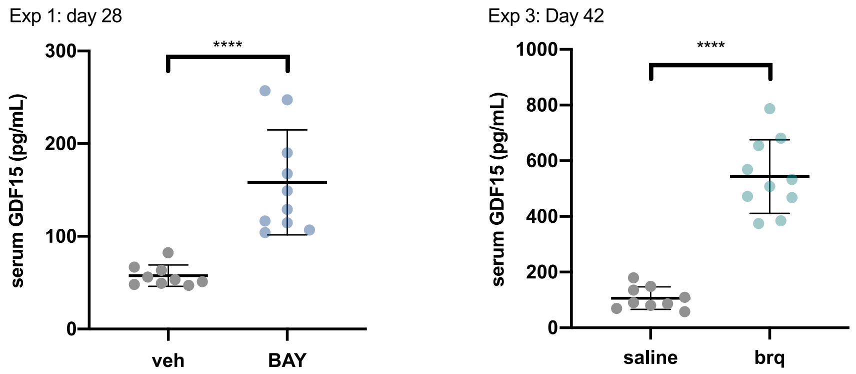


**Figure 3. DHODH inhibitors elevate circulating GDF15 in *db/db* mice**

Average (±SD) circulating GDF15 in *db/db* mice from experiment 1 (treatment started at 7 weeks of age) and experiment 3 (treatment started at 16 weeks of age). p values calculated by Student's t test.

In agreement with the results described for metformin in mice and humans (Coll et al., 2020; Day et al., 2019), a significant increase in serum GDF15 levels was noticed in young *db/db* mice treated with BAY2402234 (experiment 1) (Figure 3). A clear increase in serum GDF15 levels was also observed in older mice treated with brequinar (Figure 3, experiment 3).

## DHODH inhibitors reduce food intake and polydipsia in *db/db* mice

It is widely accepted that GDF15 influences food intake (Tsai et al., 2018). Therefore, we tested whether DHODH inhibitors also reduce appetite in *db/db* hyperphagic mice. As shown in Figure 4A, BAY2402234 as well as brequinar reduced food intake.

DHODH inhibitors did not have significant effect on body weight in young *db/db* mice (experiment 2). In agreement with previous observations (Coleman and Hummel, 1967), older *db/db* mice (experiment 3) lost weight over time (Figure 4B), but this body weight loss decelerated in mice treated with brequinar.

Aside from hyperphagia, polydipsia is a feature of *db/db* mice (Coleman and Hummel, 1967). Here we show that BAY2402234 and brequinar caused a stark reduction in water consumption in *db/db* mice, which could be detected shortly after treatment was started and before changes in food intake were noticeable (Figure 4C).

## DHODH inhibitors improve glucose control in *db/db* mice

Having in mind the effects on mitochondria, metabolism, and GDF15 we evaluated whether DHODH blockage could lower glycemia in *db/db* mice. Accordingly, and as shown in Figure 5A, brequinar improved glucose tolerance in young mice (experiment 2) and also in older mice (experiment 3). Fasting and non-fasting blood glucose levels were also lower in brequinar-treated mice than in controls (Figures 5C and S4). The improvements in blood glucose were mirrored by improvement in HbA1c levels in young mice treated with DHODH inhibitors (experiments 1 and 2) (Figures 5B and S5). In older mice treated with brequinar (experiment 3), the levels of HbA1c were clearly reduced over time (Figure 5B). Furthermore, as shown in the insulin tolerance test for experiment 3 (Figure 5D), on day 30 after treatment, that is, when mice were approximately 20 weeks old, brequinar treatment improved sensitivity to insulin.

## DHODH inhibitors delay loss of β cell mass in *db/db* mice

It is well established that at 10 days of age, *db/db* mice start to display hyperinsulinemia and between 8 and 12 weeks of age the concentration of insulin reaches a maximum of approximately 6–10 times the normal levels (0.8–1.2 ng/mL). At this time point, the total mass of pancreatic β cells gradually declines, and this is followed by an abrupt drop in blood insulin levels until death (Coleman and Hummel, 1967; Dalbøge et al., 2013; Puff et al., 2011). Accordingly, we observed that vehicle-treated *db/db* mice presented an average non-fasting serum insulin concentration of 12.11 ng/mL when they were 11 weeks old (experiment 1) and 2.05 ng/mL when they were 22 weeks old (experiment 3) (Figure 6A).

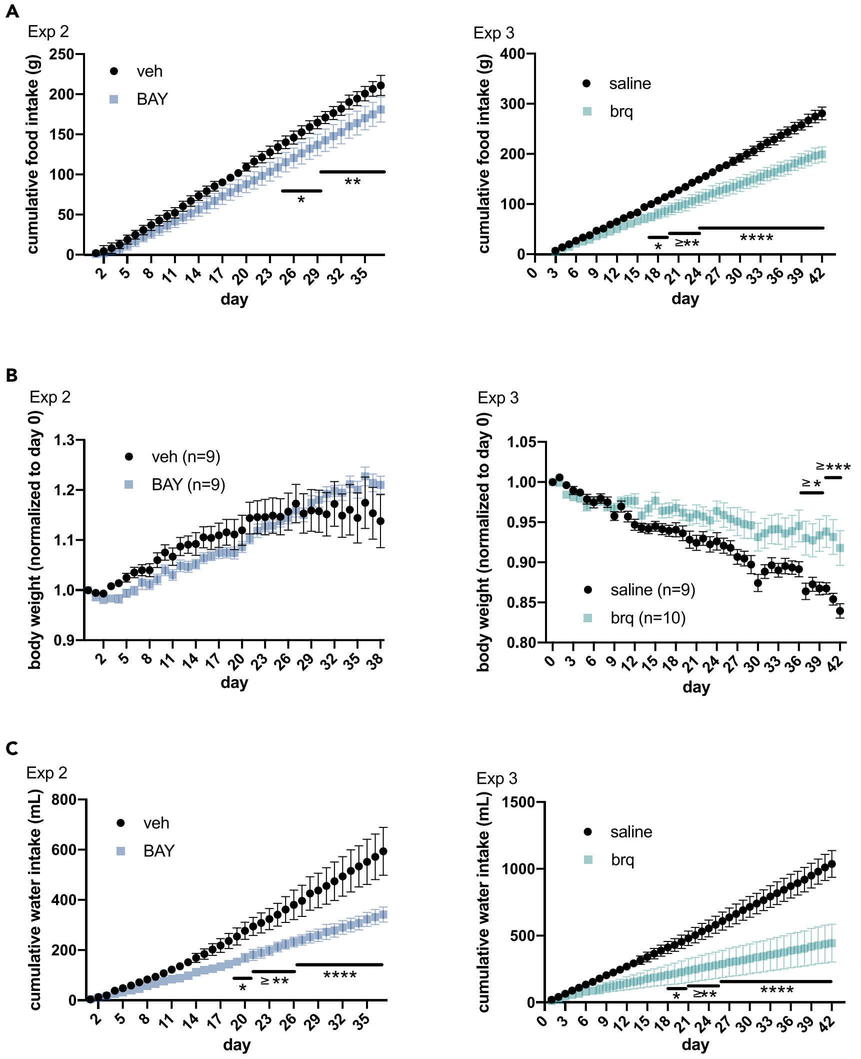


**Figure 4. DHODH inhibitors reduce food intake and polydipsia in *db/db* mice but do not affect body weight**

(A) Cumulative food intake per *db/db* mouse measured daily in experiment 2 (treatment started at 7 weeks of age) and experiment 3 (treatment started at 16 weeks of age). Values correspond to the mean (±SD) of 3 cages (n = 3). p values are calculated by two-way ANOVA.

(B) Average body weights (±SEM) normalized to the weight at the start of the experiments. p values by two-way ANOVA.

(C) Cumulative water intake per *db/db* mouse measured daily in experiments 2 and 3. Values correspond to the mean (±SD) of 3 cages (n = 3). p values by two-way ANOVA.

In experiment 1 (where 7-week-old mice were treated with BAY2402234 for 4 weeks), non-fasting serum insulin levels in treated mice were significantly higher (32.42 ng/mL) than in vehicle-treated mice (12.11 ng/mL) (Figure 6A). In experiment 3, where older mice (16 weeks old) were treated with brequinar for 6 weeks, the average non-fasting serum insulin level was 10.06 ng/mL, also significantly higher than in control-treated mice (Figure 6A). In this experiment, 2 of the 10 mice had remarkably high levels of insulin above 20 ng/mL and were also the ones that showed the lowest HbA1c values in Figure 5B. The other 8 mice presented an average non-fasting serum insulin concentration of 3.10 ng/mL, which is still significantly higher (p = 0.01) than the average value for the saline control-treated mice (2.05 ng/mL).

Subsequently, we stained the pancreas of the mice from experiments 1 and 3 with an antibody against insulin. This revealed that on average, the number of insulin-positive Langerhans islets and the area occupied by them were larger in BAY2402234- or brequinar-treated mice than in controls (Figures 6B, 6C, and S6). In the brequinar experiment with older mice (experiment 3), we also stained for insulin and activated caspase

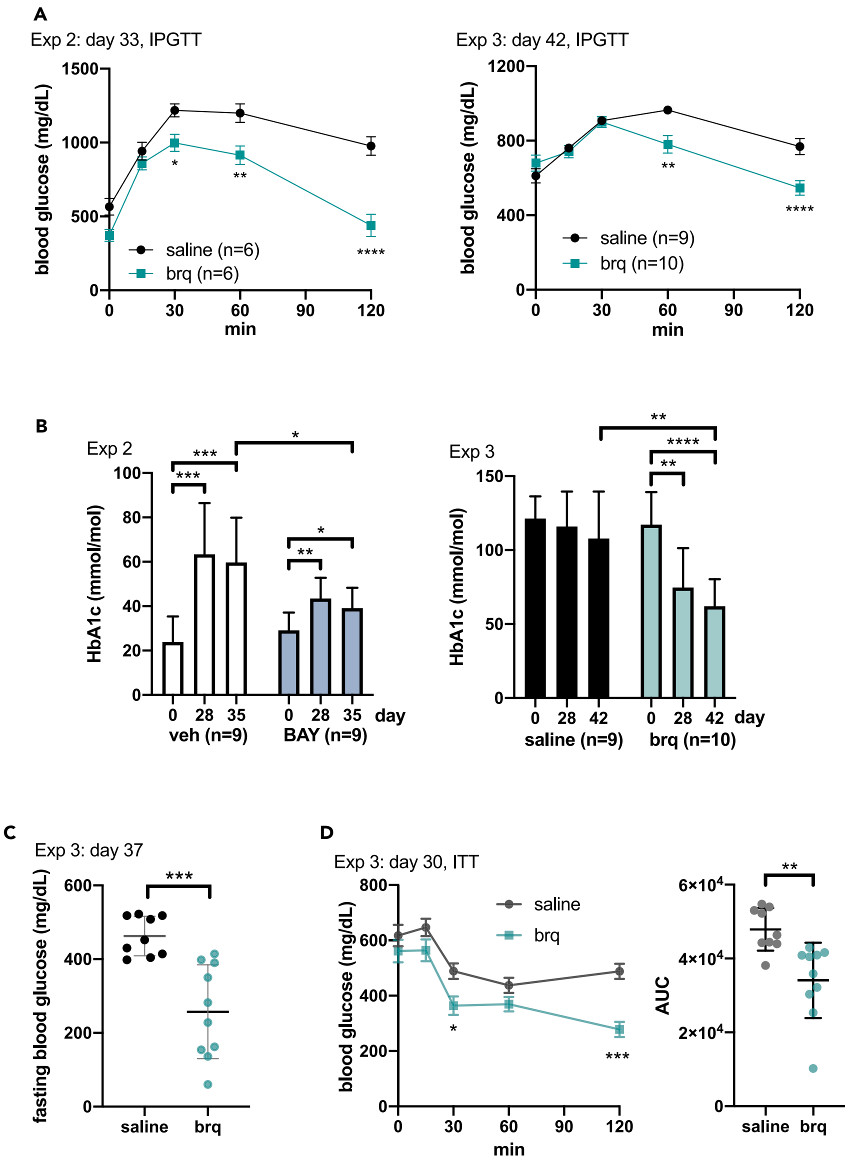


**Figure 5. DHODH inhibitors improve glucose control in *db/db* mice**

(A) Intraperitoneal glucose tolerance test (IPGTT) for experiments 2 and 3 on the indicated days after treatment start. Graph shows mean values (±SEM). p values were calculated using two-way ANOVA.

(B) Average (±SD) HbA1c levels at the indicated days after treatment in experiments 2 and 3. p values by Student's t test. In experiment 3, several measurements were above the range of the apparatus and were assigned the maximum value (130 mmol/mol).

(C) Average (±SD) fasting blood glucose in experiment 3 measured on day 37. Note that the effect of brequinar on fasting blood glucose is significant in this measurement, whereas the effect on fasting blood glucose is lost in the zero time point of the insulin tolerance test (ITT) assay in experiment 3 shown in (D). It is possible that this lack of effect is because in (D), fasting glucose was measured on day 30, which is 3 days after brequinar administration, whereas day 37 is only 1 day after the injection of brequinar. Supporting this view, the effect of brequinar on non-fasting blood glucose levels is only significant on the day after injection (Figure S4). p values obtained using the Student's t test.

(D) Insulin sensitivity test for experiment 3. Left graph shows mean values (±SEM) and p values calculated using two-way ANOVA. Right graph shows area under the curve for each mouse. Error bars indicate SD, and p values are calculated by Student's t test.

See also Figures S4 and S5.

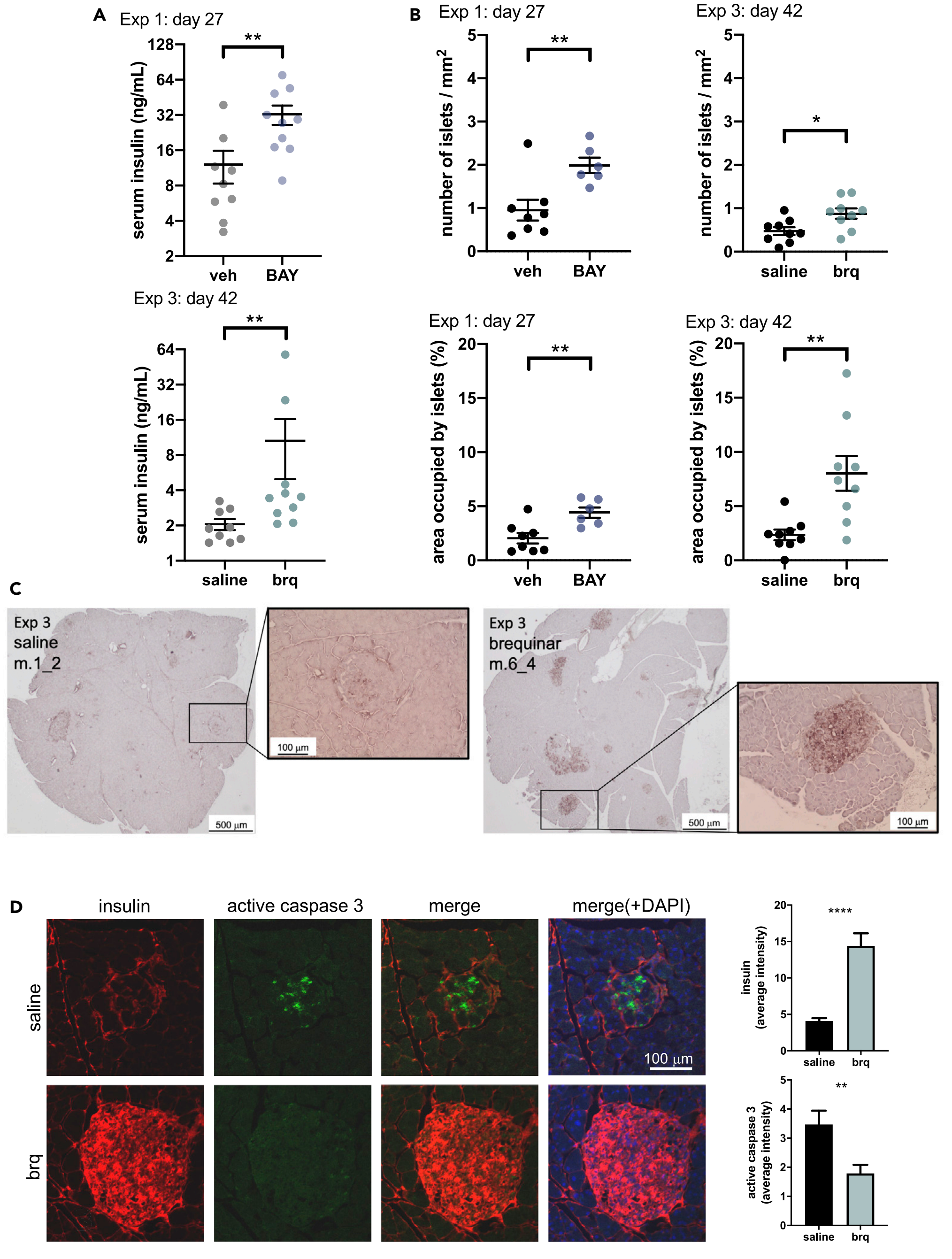


**Figure 6. DHODH inhibitors prevent β cell mass loss in *db/db* mice**

(A) Average (±SEM) serum insulin levels in mice from experiment 1 (treatment start at 7 weeks of age) and experiment 3 (treatment start at 16 weeks of age) after 27 and 42 days of treatment with the indicated DHODH inhibitor, respectively. p values calculated by Mann-Whitney's test.

(B) Insulin-positive islet numbers and area occupied by these islets measured in experiments 1 and 3. Error bars show SEM, and p values are calculated by Student's t test.

(C) Representative staining of pancreases from experiment 3 (mice 1_2 and 6_4) with an antibody against insulin.

(D) Representative staining of islets with antibodies against insulin and activated caspase 3 in pancreases from experiment 3. Bar graphs show average counts (±SEM) and p values are calculated by Student's t test. n = 23 islets for saline control, and n = 26 islets for brequinar treated.

See also Figure S6.

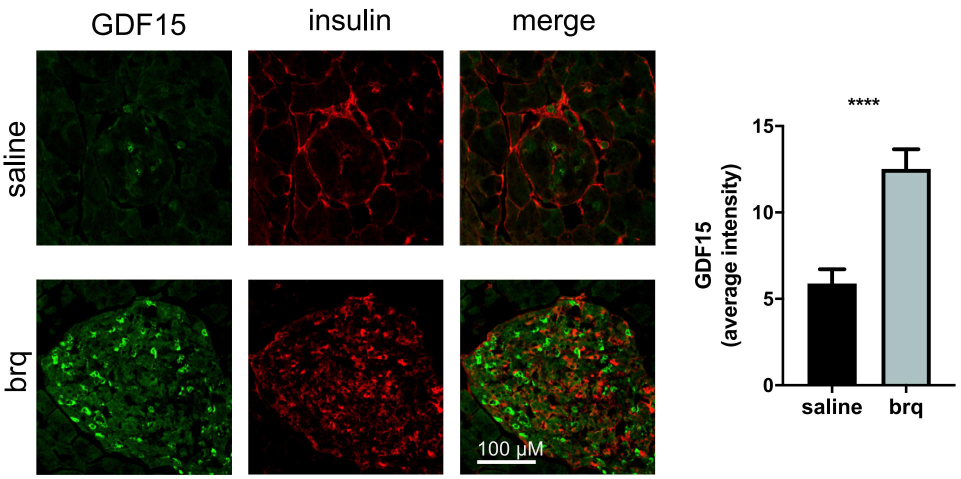


**Figure 7. DHODH inhibitors increase GDF15 expression in pancreatic islets**

Representative staining of islets with antibodies against GDF15 and insulin in pancreases from experiment 3. Bar graphs show average counts (±SEM), and p values are calculated by Student's t test. n = 28 islets for saline control, and n = 25 islets for brequinar treated.

3 as a marker for apoptosis. As shown in Figure 6D, insulin labeling was more intense in brequinar-treated mice than in controls and this correlated with a weaker signal for activated caspase 3. Altogether, these analyses suggest that administration of DHODH inhibitors delays β cell loss in *db/db* mice.

Interestingly, a significant increase in GDF15 levels was detected in pancreatic islets from mice treated with DHODH inhibitors (Figure 7). Of note, this increase was only evident in the cells with less insulin.

## DISCUSSION

The results presented in this work demonstrate that pharmacological inhibition of DHODH improves glucose balance in hyperphagic *db/db* mice. This associates with the ability of DHODH inhibitors to reduce mitochondrial respiration, induce GDF15 expression and circulating GDF15, reduce food and water intake, and protect β cells from apoptosis.

In addition to reducing food consumption, GDF15 is anorectic (Tsai et al., 2018) and mediates the body weight loss induced by metformin (Coll et al., 2020; Day et al., 2019). However, DHODH inhibitors neither reduced the rate of body weight gain in younger *db/db* mice nor enhanced weight loss in the older *db/db* mice, despite the observed reduction in food consumption. Instead, DHODH inhibitors, and especially clearly in the experiment with older mice, protected from body weight loss. Insulin treatment is associated with increased body weight in humans and as previously reported, also in *db/db* mice (Sugizaki et al., 2017). Here we show that DHODH inhibitor-treated *db/db* mice have higher levels of serum insulin than controls and that their β cells are protected from apoptosis. Therefore, this increase in insulin production could explain why DHODH-treated mice did not lose weight.

In summary, this study suggests a model (Figure 8) wherein DHODH inhibitors reduce mitochondrial respiration to a moderate extent, but perhaps sufficiently to promote glucose consumption by tissues and reduce hyperglycemia. In addition, by depleting pyrimidine ribonucleotide pools, DHODH inhibitors activate p53, which in turn enhances the expression of GDF15. Increased circulating GDF15 could reduce appetite, thus delaying hyperglycemia and β cell exhaustion in *db/db* mice.

Whether type 2 diabetes is an autoimmune disease is still a matter of debate (de Candia et al., 2019; Tsai et al., 2015). Furthermore, the literature on the importance of the immune system on the development of diabetes in *db/db* mice suggests that the immune system plays a very small role in pancreatic β cell death in this mouse model (Leiter et al., 1987). However, because GDF15 can protect the pancreas from inflammation at least in type 1 diabetes (Nakayasu et al., 2020), and because DHODH inhibitors are used against autoimmune diseases, it is still possible that to some extent, DHODH inhibitors could protect the pancreas from inflammation.

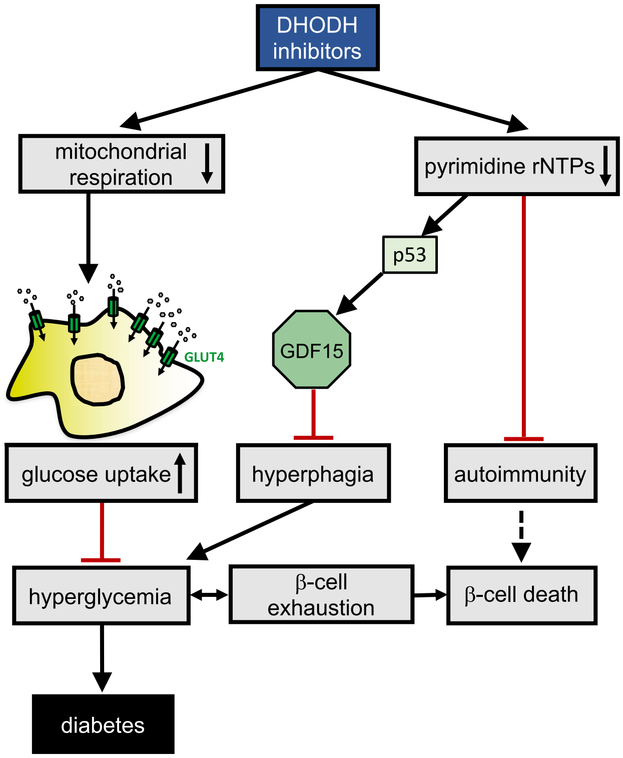


**Figure 8. Mechanistic model**
See discussion section for a description.

Hence, it could be interesting to evaluate whether leflunomide- and teriflunomide-treated patients are less prone to develop type 2 diabetes. With regard to the use of DHODH inhibitors for the treatment of COVID-19, it is also relevant to mention that diabetes is a clear risk factor. Further studies are needed to establish whether DHODH inhibitors, aside from being of use for the treatment of chronic diseases such as rheumatoid arthritis or multiple sclerosis, may be considered as antidiabetic drugs. In this regard, one advantage of pharmacological DHODH inhibition is that a wide variety of chemicals are available, some of which are extremely potent and specific as well as orally available. The structures of these chemicals are diverse and therefore, bound to have different pharmacokinetic properties. This feature may enable designing treatments for each type of disease and patient. Indeed, the success of metformin in the clinic is likely to be due to its adequate pharmacokinetic properties when compared with other biguanides (Pernicova and Korbonits, 2014). Taking into account DHODH polymorphisms is also advisable because these are known to influence patient responses to leflunomide (Grabar et al., 2009; O'Doherty et al., 2012).

## Limitations of the study

From a mechanistic point of view, confirming the model proposed in this work requires experiments using p53 knockout *db/db* mice. This will allow establishing unequivocally the role of p53 on the observed effects of DHODH inhibitors *in vivo*. Also, from a mechanistic perspective, it is still unclear whether the effect of the DHODH inhibitors on mitochondrial respiration contributes to the activation of p53 and/or to the enhancement of GDF15 expression. Again, experiments using p53 knockout *db/db* mice will be necessary to design approaches to answer this question.

## STAR★METHODS

Detailed methods are provided in the online version of this paper and include the following:

- KEY RESOURCES TABLE
- RESOURCE AVAILABILITY
  - Lead contact
  - Materials availability

- Data and code availability
- EXPERIMENTAL MODEL AND SUBJECT DETAILS
  - db/db mice
  - Cell lines
- METHOD DETAILS
  - Measurement of glucose in cell culture medium
  - GLUT4 translocation measurement
  - Cellular respiration and glycolysis
  - Western blotting
  - Measurement of GDF15 in cell culture medium
  - Experiments in db/db mice
  - Pancreas staining
- QUANTIFICATION AND STATISTICAL ANALYSIS


## SUPPLEMENTAL INFORMATION

Supplemental information can be found online at https://doi.org/10.1016/j.isci.2021.102494.


## ACKNOWLEDGMENTS

We would like to thank Madhurendra Singh and Yihai Cao for MCF7/MCF7 p53KO and 3T3-L1 cells, respectively. We also thank Nabil Djouder for critical reading and fruitful discussions. Histology and microscopy studies were performed using equipment from the Histocore and BIC facilities at Karolinska Institutet.

S.D., J.Z., G.T., H.M., D.L., M.J.G.W.L. M.A.-H., and S.L. were supported by project grants from the Swedish Research Council, the Swedish Cancer Society, and the Swedish Childhood Cancer Foundation. M.P. and E.M. were supported by the Norwegian Cancer Society (182735, 732200), Helse Vest (911884, 911789), and The Research Council of Norway through its Centers of excellence funding scheme (223250, 262652). S.-B.C. and J.G. were supported by the Swedish Research Council, Stockholm County, Research Council, Bert von Kantzows Foundation, Konung Gustaf V:s och Drottning Victorias Frimurarestifelse, and Strategic Research Programme in Diabetes. M.E.R. was supported by the Swedish Research Council grant no 2019-01691/2020-23 and Swedish Heart and Lung Foundation grant no 2018-20/20170491.


## AUTHOR CONTRIBUTIONS

Conceptualization: S.D, J.Z., and S.L.; methodology: S.D., J.Z., and G.T.; validation: M.J.G.W.L., D.L., M.P., and E.M.; formal analysis: S.D, J.Z. G.T., and S.L.; investigation: S.D., J.Z., G.T., and H.M.; resources: M.E.R., J.G., S.B.C., E.M., and M.A.-H.; writing – original draft: S.D., J.Z., S.L., and S.B.C.; writing – review & editing: S.D., J.Z., G.T., M.A.-H., E.M., M.E.R., S.B.C., and S.L.; visualization: S.D, J.Z. G.T., and S.L.; supervision: M.A.-H., E.M., M.E.R., S.B.C., and S.L.; project administration: S.L.; funding acquisition: M.A.-H., E.M., M.E.R., S.-B.C., and S.L.

## DECLARATION OF INTERESTS

S.L. is co-founder and shareholder of a new company that owns a patent on DHODH inhibitors. These inhibitors are different from the ones used in this publication. All other authors declare no competing interests.


Received: January 14, 2021
Revised: March 25, 2021
Accepted: April 27, 2021
Published: May 21, 2021


## REFERENCES


Alcorn, N., Saunders, S., and Madhok, R. (2009). Benefit-risk assessment of leflunomide: an appraisal of leflunomide in rheumatoid arthritis 10 years after licensing. Drug Saf. *32*, 1123–1134.

Becker, C., Sevilla, L., Tomàs, E., Palacin, M., Zorzano, A., and Fischer, Y. (2001). The endosomal compartment is an insulin-sensitive recruitment site for GLUT4 and GLUT1 glucose transporters in cardiac myocytes. Endocrinology *142*, 5267–5276.

Boukalova, S., Hubackova, S., Milosevic, M., Ezrova, Z., Neuzil, J., and Rohlena, J. (2020). Dihydroorotate dehydrogenase in oxidative phosphorylation and cancer. Biochim. Biophys. Acta Mol. Basis Dis. *1866*, 165759.

Brent, R.L. (2001). Teratogen update: reproductive risks of leflunomide (arava); A pyrimidine synthesis inhibitor: counseling women

taking leflunomide before or during pregnancy and men taking leflunomide who are contemplating fathering a child. Teratology *63*, 106–112.

Bridges, H.R., Sirviö, V.A., Agip, A.-N.A., and Hirst, J. (2016). Molecular features of biguanides required for targeting of mitochondrial respiratory complex I and activation of AMP-kinase. BMC Biol. *14*, 65.

Brown, K.K., Spinelli, J.B., Asara, J.M., and Toker, A. (2017). Adaptive reprogramming of de novo pyrimidine synthesis is a metabolic vulnerability in triple-negative breast cancer. Cancer Discov. *7*, 391–399.

de Candia, P., Prattichizzo, F., Garavelli, S., De Rosa, V., Galgani, M., Di Rella, F., Spagnuolo, M.I., Colamatteo, A., Fusco, C., Micillo, T., et al. (2019). Type 2 diabetes: how much of an autoimmune disease? Front. Endocrinol. *10*, 451.

Christian, S., Merz, C., Evans, L., Gradl, S., Seidel, H., Friberg, A., Eheim, A., Lejeune, P., Brzezinka, K., Zimmermann, K., et al. (2019). The novel dihydroorotate dehydrogenase (DHODH) inhibitor BAY 2402234 triggers differentiation and is effective in the treatment of myeloid malignancies. Leukemia *33*, 2403–2415.

Coleman, D.L., and Hummel, K.P. (1967). Studies with the mutation, diabetes, in the mouse. Diabetologia *3*, 238–248.

Coll, A.P., Chen, M., Taskar, P., Rimmington, D., Patel, S., Tadross, J.A., Cimino, I., Yang, M., Welsh, P., Virtue, S., et al. (2020). GDF15 mediates the effects of metformin on body weight and energy balance. Nature *578*, 444–448.

Dalbøge, L.S., Almholt, D.L.C., Neerup, T.S.R., Vassiliadis, E., Vrang, N., Pedersen, L., Fosgerau, K., and Jelsing, J. (2013). Characterisation of age-dependent beta cell dynamics in the male db/db mice. PLoS One *8*, e82813.

Day, E.A., Ford, R.J., Smith, B.K., Mohammadi-Shemirani, P., Morrow, M.R., Gutgesell, R.M., Lu, R., Raphenya, A.R., Kabiri, M., McArthur, A.G., et al. (2019). Metformin-induced increases in GDF15 are important for suppressing appetite and promoting weight loss. Nat. Metab. *1*, 1202–1208.

Deans, R.M., Morgens, D.W., Ökesli, A., Pillay, S., Horlbeck, M.A., Kampmann, M., Gilbert, L.A., Li, A., Mateo, R., Smith, M., et al. (2016). Parallel shRNA and CRISPR-Cas9 screens enable antiviral drug target identification. Nat. Chem. Biol. *12*, 361–366.

Doscas, M.E., Williamson, A.J., Usha, L., Bogachkov, Y., Rao, G.S., Xiao, F., Wang, Y., Ruby, C., Kaufman, H., Zhou, J., et al. (2014). Inhibition of p70 S6 kinase (S6K1) activity by A77 1726 and its effect on cell proliferation and cell cycle progress. Neoplasia N. Y. N. *16*, 824–834.

Fang, J., Uchiumi, T., Yagi, M., Matsumoto, S., Amamoto, R., Takazaki, S., Yamaza, H., Nonaka, K., and Kang, D. (2013). Dihydro-orotate dehydrogenase is physically associated with the respiratory complex and its loss leads to mitochondrial dysfunction. Biosci. Rep. *33*, e00021.

Gattermann, N., Dadak, M., Hofhaus, G., Wulfert, M., Berneburg, M., Loeffler, M.L., and Simmonds, H.A. (2004). Severe impairment of nucleotide synthesis through inhibition of mitochondrial respiration. Nucleosides Nucleotides Nucleic Acids *23*, 1275–1279.

Gerstein, H.C., Pare, G., Hess, S., Ford, R.J., Sjaarda, J., Raman, K., McQueen, M., Lee, S., Haenel, H., Steinberg, G.R., et al. (2017). Growth differentiation factor 15 as a novel biomarker for metformin. Diabetes Care *40*, 280–283.

Grabar, P.B., Rozman, B., Logar, D., Praprotnik, S., and Dolzan, V. (2009). Dihydroorotate dehydrogenase polymorphism influences the toxicity of leflunomide treatment in patients with rheumatoid arthritis. Ann. Rheum. Dis. *68*, 1367–1368.

Khutornenko, A.A., Roudko, V.V., Chernyak, B.V., Vartapetian, A.B., Chumakov, P.M., and Evstafieva, A.G. (2010). Pyrimidine biosynthesis links mitochondrial respiration to the p53 pathway. Proc. Natl. Acad. Sci. U. S. A. *107*, 12828–12833.

Ladds, M.J.G.W., van Leeuwen, I.M.M., Drummond, C.J., Chu, S., Healy, A.R., Popova, G., Pastor Fernández, A., Mollick, T., Darekar, S., Sedimbi, S.K., et al. (2018). A DHODH inhibitor increases p53 synthesis and enhances tumor cell killing by p53 degradation blockage. Nat. Commun. *9*, 1107.

Leiter, E.H., Prochazka, M., and Shultz, L.D. (1987). Effect of immunodeficiency on diabetogenesis in genetically diabetic (db/db) mice. J. Immunol. Baltim. Md. *138*, 3224–3229.

Li, P.X., Wong, J., Ayed, A., Ngo, D., Brade, A.M., Arrowsmith, C., Austin, R.C., and Klamut, H.J. (2000). Placental transforming growth factor-beta is a downstream mediator of the growth arrest and apoptotic response of tumor cells to DNA damage and p53 overexpression. J. Biol. Chem. *275*, 20127–20135.

Lim, C.-Y., Bi, X., Wu, D., Kim, J.B., Gunning, P.W., Hong, W., and Han, W. (2015). Tropomodulin3 is a novel Akt2 effector regulating insulin-stimulated GLUT4 exocytosis through cortical actin remodeling. Nat. Commun. *6*, 5951.

Löffler, M., Jöckel, J., Schuster, G., and Becker, C. (1997). Dihydroorotat-ubiquinone oxidoreductase links mitochondria in the biosynthesis of pyrimidine nucleotides. Mol. Cell. Biochem. *174*, 125–129.

Lu, X., Burbidge, S.A., Griffin, S., and Smith, H.M. (1996). Discordance between accumulated p53 protein level and its transcriptional activity in response to u.v. radiation. Oncogene *13*, 413–418.

Madiraju, A.K., Erion, D.M., Rahimi, Y., Zhang, X.-M., Braddock, D.T., Albright, R.A., Prigaro, B.J., Wood, J.L., Bhanot, S., MacDonald, M.J., et al. (2014). Metformin suppresses gluconeogenesis by inhibiting mitochondrial glycerophosphate dehydrogenase. Nature *510*, 542–546.

Matheu, A., Maraver, A., Klatt, P., Flores, I., Garcia-Cao, I., Borras, C., Flores, J.M., Viña, J., Blasco, M.A., and Serrano, M. (2007). Delayed ageing through damage protection by the Arf/p53 pathway. Nature *448*, 375–379.

Mathur, D., Stratikopoulos, E., Ozturk, S., Steinbach, N., Pegno, S., Schoenfeld, S., Yong, R., Murty, V.V., Asara, J.M., Cantley, L.C., et al. (2017). PTEN regulates glutamine flux to pyrimidine synthesis and sensitivity to dihydroorotate dehydrogenase inhibition. Cancer Discov. *7*, 380–390.

Mazagova, M., Buikema, H., van Buiten, A., Duin, M., Goris, M., Sandovici, M., Henning, R.H., and Deelman, L.E. (2013). Genetic deletion of growth differentiation factor 15 augments renal damage in both type 1 and type 2 models of diabetes. Am. J. Physiol. Ren. Physiol. *305*, F1249–F1264.

Miret-Casals, L., Sebastián, D., Brea, J., Rico-Leo, E.M., Palacín, M., Fernández-Salguero, P.M., Loza, M.I., Albericio, F., and Zorzano, A. (2018). Identification of new activators of mitochondrial fusion reveals a link between mitochondrial morphology and pyrimidine metabolism. Cell Chem. Biol. *25*, 268–278.e4.

Mollick, T., and Laín, S. (2020). Modulating pyrimidine ribonucleotide levels for the treatment of cancer. Cancer Metab. *8*, 12.

Mullican, S.E., Lin-Schmidt, X., Chin, C.-N., Chavez, J.A., Furman, J.L., Armstrong, A.A., Beck, S.C., South, V.J., Dinh, T.Q., Cash-Mason, T.D., et al. (2017). GFRAL is the receptor for GDF15 and the ligand promotes weight loss in mice and nonhuman primates. Nat. Med. *23*, 1150–1157.

Munier-Lehmann, H., Vidalain, P.-O., Tangy, F., and Janin, Y.L. (2013). On dihydroorotate dehydrogenases and their inhibitors and uses. J. Med. Chem. *56*, 3148–3167.

Nakayasu, E.S., Syed, F., Tersey, S.A., Gritsenko, M.A., Mitchell, H.D., Chan, C.Y., Dirice, E., Turatsinze, J.-V., Cui, Y., Kulkarni, R.N., et al. (2020). Comprehensive proteomics analysis of stressed human islets identifies GDF15 as a target for type 1 diabetes intervention. Cell Metab *31*, 363–374.e6.

O'Doherty, C., Schnabl, M., Spargo, L., Cleland, L.G., James, M., Proudman, S.M., and Wiese, M.D. (2012). Association of DHODH haplotype variants and response to leflunomide treatment in rheumatoid arthritis. Pharmacogenomics *13*, 1427–1434.

Owen, M.R., Doran, E., and Halestrap, A.P. (2000). Evidence that metformin exerts its anti-diabetic effects through inhibition of complex 1 of the mitochondrial respiratory chain. Biochem. J. *348*, 607–614.

Patel, S., Alvarez-Guaita, A., Melvin, A., Rimmington, D., Dattilo, A., Miedzybrodzka, E.L., Cimino, I., Maurin, A.-C., Roberts, G.P., Meek, C.L., et al. (2019). GDF15 provides an endocrine signal of nutritional stress in mice and humans. Cell Metab *29*, 707–718.e8.

Pernicova, I., and Korbonits, M. (2014). Metformin–mode of action and clinical implications for diabetes and cancer. Nat. Rev. Endocrinol. *10*, 143–156.

Popova, G., Ladds, M.J.G.W., Johansson, L., Saleh, A., Larsson, J., Sandberg, L., Sahlberg, S.H., Qian, W., Gullberg, H., Garg, N., et al. (2020). Optimization of tetrahydroindazoles as inhibitors of human dihydroorotate dehydrogenase and evaluation of their activity

and in vitro metabolic stability. J. Med. Chem. *63*, 3915–3934.

Puff, R., Dames, P., Weise, M., Göke, B., Seissler, J., Parhofer, K.G., and Lechner, A. (2011). Reduced proliferation and a high apoptotic frequency of pancreatic beta cells contribute to genetically-determined diabetes susceptibility of db/db BKS mice. Horm. Metab. Res. Horm. Stoffwechselforschung Horm. Metab. *43*, 306–311.

Rawls, J., Knecht, W., Diekert, K., Lill, R., and Löffler, M. (2000). Requirements for the mitochondrial import and localization of dihydroorotate dehydrogenase. Eur. J. Biochem. *267*, 2079–2087.

Riscal, R., Schrepfer, E., Arena, G., Cissé, M.Y., Bellvert, F., Heuillet, M., Rambow, F., Bonneil, E., Sabourdy, F., Vincent, C., et al. (2016). Chromatin-bound MDM2 regulates serine metabolism and redox homeostasis independently of p53. Mol. Cell *62*, 890–902.

Sugizaki, T., Zhu, S., Guo, G., Matsumoto, A., Zhao, J., Endo, M., Horiguchi, H., Morinaga, J., Tian, Z., Kadomatsu, T., et al. (2017). Treatment of diabetic mice with the SGLT2 inhibitor TA-1887 antagonizes diabetic cachexia and decreases mortality. NPJ Aging Mech. Dis. *3*, 12.

Sykes, D.B., Kfoury, Y.S., Mercier, F.E., Wawer, M.J., Law, J.M., Haynes, M.K., Lewis, T.A., Schajnovitz, A., Jain, E., Lee, D., et al. (2016). Inhibition of dihydroorotate dehydrogenase overcomes differentiation blockade in acute myeloid leukemia. Cell *167*, 171–186.e15.

Tsai, S., Clemente-Casares, X., Revelo, X.S., Winer, S., and Winer, D.A. (2015). Are obesity-related insulin resistance and type 2 diabetes autoimmune diseases? Diabetes *64*, 1886–1897.

Tsai, V.W.W., Husaini, Y., Sainsbury, A., Brown, D.A., and Breit, S.N. (2018). The MIC-1/GDF15-GFRAL pathway in energy homeostasis: implications for obesity, cachexia, and other associated diseases. Cell Metab *28*, 353–368.

Wang, X., Chrysovergis, K., Kosak, J., Kissling, G., Streicker, M., Moser, G., Li, R., and Eling, T.E. (2014). hNAG-1 increases lifespan by regulating energy metabolism and insulin/IGF-1/mTOR signaling. Aging *6*, 690–704.

Vélez, J., Hail, N., Jr., Konopleva, M., Zeng, Z., Kojima, K., Samudio, I., and Andreeff, M. (2013). Mitochondrial uncoupling and the reprograming of intermediary metabolism in leukemia cells. Front. Oncol. *3*, 67.

Wang, Y., An, H., Liu, T., Qin, C., Sesaki, H., Guo, S., Radovick, S., Hussain, M., Maheshwari, A., Wondisford, F.E., et al. (2019). Metformin improves mitochondrial respiratory activity through activation of AMPK. Cell Rep *29*, 1511–1523.e5.

White, R.M., Cech, J., Ratanasirintrawoot, S., Lin, C.Y., Rahl, P.B., Burke, C.J., Langdon, E., Tomlinson, M.L., Mosher, J., Kaufman, C., et al. (2011). DHODH modulates transcriptional elongation in the neural crest and melanoma. Nature *471*, 518–522.

Xiong, R., Zhang, L., Li, S., Sun, Y., Ding, M., Wang, Y., Zhao, Y., Wu, Y., Shang, W., Jiang, X., et al. (2020). Novel and potent inhibitors targeting DHODH, a rate-limiting enzyme in de novo pyrimidine biosynthesis, are broad-spectrum antiviral against RNA viruses including newly emerged coronavirus SARS-CoV-2. BioRxiv.

Zhu, J., Singh, M., Selivanova, G., and Peuget, S. (2020). Pifithrin-α alters p53 post-translational modifications pattern and differentially inhibits p53 target genes. Sci. Rep. *10*, 1049.

# STAR★METHODS

## KEY RESOURCES TABLE

| REAGENT or RESOURCE | SOURCE | IDENTIFIER |
|---|---|---|
| Antibodies | | |
| Insulin mouse monoclonal antibody (2D11-H5) | Santa Cruz Biotechnology | Cat#sc-8033; RRID:AB_627285 |
| Cleaved Caspase 3 rabbit monoclonal antibody | Cell Signaling Technology | Cat#9664; RRID:AB_2070042 |
| c-Myc mouse monoclonal antibody (9E10) | Thermo Fisher Scientific | Cat#13-2500; RRID:AB_2533008 |
| GDF15 mouse monoclonal antibody | Sigma-Aldrich | Cat#AMAB90687; RRID:AB_2665632 |
| GDF15/MIC-1 Polyclonal Antibody | Bioss | Cat#BS-3818R; RRID:AB_10857699 |
| p53 mouse monoclonal antibody DO1 | Abcam | Cat#ab1101; RRID:AB_297667 |
| Rabbit monoclonal antibody to ATF4 (EPR18111) | Abcam | Cat#ab184909; RRID:AB_2819059 |
| Rabbit polyclonal antibody to Histone H3 | Abcam | Cat#ab1791; RRID:AB_302613 |
| Alexa Fluor Plus 488 secondary antibody | Thermo Fisher Scientific | Cat#A32731; RRID:AB_2633280 |
| Alexa Fluor Plus 555 secondary antibody | Thermo Fisher Scientific | Cat#A32727; RRID:AB_2633276 |
| Chemicals, peptides, and recombinant proteins | | |
| Brequinar | Sigma-Aldrich<br>Bio-techne | Cat#SML0113<br>*6196* |
| BAY2402234 | Med Chem Express | Cat#HY-112645 |
| Metformin | Selleckchem | Cat#S1950 |
| Insulin Novorapid | Novo Nordisk | Cat#A10AB05 |
| D-glucose | Merck | Cat#G8270 |
| Uridine | Alfa Aesar (Thermofisher scientific) | Cat#A15227.14 |
| Critical commercial assays | | |
| Glucose Quantification Kit | Sigma-Aldrich | Cat#MAK263 |
| Seahorse XF base medium | Agilent Technologies | Cat#103335-100 |
| Seahorse XF Glycolysis Stress Test Kit | Agilent Technologies | Cat#103020-100 |
| Mouse/Rat GDF15 Quantikine ELISA Kit | R&D Systems | Cat#MGD150 |
| Human GDF15 Quantikine ELISA Kit | R&D Systems | Cat#DGD150 |
| DC protein assay | Bio-Rad | Cat#5000112 |
| Mouse insulin ELISA kit | Mercodia | Cat#10-1247-01; RRID:AB_2783837 |
| IHC Select Immunoperoxidase Secondary Detection | Millipore | Cat#DAB150 |
| Experimental models: Cell lines | | |
| MCF7 and MCF7 p53KO | Previously described (*Zhu et al., 2020*) | NA |
| T22-RGCΔFos-LacZ murine fibroblasts | Previously described (Lu et al., 1996) | NA |
| 3T3-L1 adipocytes | Professor Yihai Cao (Karolinska Institutet) | NA |
| Experimental models: Organisms/strains | | |
| Mouse: BKS(D)-Leprdb/JOrlRj (db/db) | Janvier | Cat#SM-DB-F |
| Recombinant DNA | | |
| pLenti-myc-GLUT4-mCherry | Addgene | Cat#64049; RRID:Addgene_64049 |
| Software and algorithms | | |
| Fiji (Image J) | NIH | https://imagej.net/Fiji/Downloads |

(*Continued on next page*)

*Continued*

| REAGENT or RESOURCE | SOURCE | IDENTIFIER |
|---|---|---|
| Wave2.6 | Agilent Technologies | https://www.agilent.com/en/product/cell-analysis/real-time-cell-metabolic-analysis/xf-software/seahorse-wave-desktop-software-740897 |
| ZEN2.3 | Zeiss | https://www.zeiss.com/microscopy/int/products/microscope-software/zen.html |
| Deposited data | | |
| Western blott data | This paper | Mendeley data (https://doi.org/10.17632/76742x9w87.1) |
| Animal experiments data | This paper | Mendeley data (https://doi.org/10.17632/76742x9w87.1) |
| Other | | |
| Accucheck Aviva strips | Roche | https://www.accu-chek.se/blodsockermatare/aviva |
| Prima Home Test Multicare-IN Glucometer | BSI | 895698 |
| DCA vantage analyzer for HbA1c | Siemens | https://www.siemens-healthineers.com/diabetes/diabetes/dca-vantage-analyzer |

## RESOURCE AVAILABILITY

### Lead contact

Information and requests for resources should be directed to and will be fulfilled by the Lead Contact, Suhas Darekar (suhas.drarekar@ki.se).

### Materials availability

This study did not generate new unique reagents.

### Data and code availability

The published article contains all data generated or analyzed.

Original/source data for figures in the paper is available on Mendeley Data.

http://dx.doi.org/10.17632/76742x9w87.2

## EXPERIMENTAL MODEL AND SUBJECT DETAILS

### db/db mice

7 week old female BKS(D)-Leprdb/JOrlRj (*db/db*) obese leptin receptor deficient mice on C57BLKS/J (BKS) genetic background were purchased (SM-DB-F, Janvier, Le Genest St. Isle, France). Mice were housed under specific pathogen-free conditions and according to directives and guidelines of the Swedish Board of Agriculture, the Swedish Animal Protection Agency, and Karolinska Institutet. Animals were kept under controlled light (12 h:12 h light:dark cycle), 21-22°C and 50 $\pm$ 20% humidity and had access to standard chow diet containing 28,7 protein kcal%, 13,4 fat kcal% and 57,9 carbohydrate kcal% and water *ad libitum*. Animals were quarantined and acclimated for at least 5 days before starting an experiment.

Experimental protocols were approved by the regional ethical board (Stockholms djurförsöksetiska nämnd) in accordance to national regulations outlined in L150 (Föreskrifter och allmänna råd om Försöksdjur, SJVFS 2012:26). During treatments, mice were monitored daily for signs of morbidity and body weight loss. Food and water intake were also monitored every day by weighing the crates containing the chow pellets and the water bottles in each cage. Mice were randomly distributed into cages (3-4 mice per cage). Researchers were not blinded to group allocations.

### Cell lines

In this work we used human breast cancer cells MCF7 and MCF7 p53KO and murine cell lines T22-RGCΔFos-LacZ fibroblasts and 3T3-L1 adipocytes. Cell lines were not authenticated. For references on their origin see key resources table.

## METHOD DETAILS

### Measurement of glucose in cell culture medium

Murine T22-RGCΔFos-LacZ cells (previously described(Lu et al., 1996)) were seeded at 5 x $10^4$ cells/well of a 6-well plate in DMEM medium from Sigma-Aldrich (high glucose) supplemented with 10% fetal bovine serum (FBS) and 1% penicillin/streptomycin. Next day, medium was replaced with complete McCoy's 5A medium (3000 ng/μL glucose and 219.15 ng/μL L-Glutamine supplemented with 10% FBS and 1% penicillin/streptomycin, Sigma-Aldrich) containing vehicle control or test compounds. 72 h after treatment with brequinar (Bio-techne, 6196), glucose content in the cell culture medium was determined with the Prima Home Test Multicare-IN Glucometer (BSI, 895698).

### GLUT4 translocation measurement

3T3-L1 adipocytes (Kind gift from Professor Yihai Cao, Karolinska Institutet) were transfected with a plasmid (Addgene, 64049) expressing GLUT4 fusion protein (Myc-GLUT4-mCherry) as described (Lim et al., 2015). 24 hours after transfection, cells were treated with 100 nM BAY2402234 (Med Chem Express, HY-112645), 1 μM brequinar (Bio-techne, 6196) or 2 mM metformin (Selleckchem, S1950) for 24 hours and then fixed with 4% paraformaldehyde. As a positive control, cells were treated with 200 nM insulin (Sigma-Aldrich, I9278) for 20 min after a 3 h serum starvation. In non-permeabilized conditions, cells were incubated with primary mouse anti-Myc antibody (9E10, Thermo Fisher Scientific, 13-2500) followed by Alexa Fluor 488-conjugated anti-mouse secondary antibody (Thermo Fisher Scientific, A32723). Mounted samples were subjected to confocal imaging. Fiji software (ImageJ, NIH) was used for quantitative measurements of GLUT4. The ratio of surface to total GLUT4 was quantified by measuring anti-Myc fluorescence immunolabelling and mCherry fluorescence.

### Cellular respiration and glycolysis

Cellular respiration and glycolysis were measured by Seahorse XFe96 Extracellular Flux Analyzer (Agilent Technologies, Santa Clara, CA, USA). Briefly, T22-ΔFosRGC-LacZ cells were seeded at a density of 2,500 per well in Seahorse XF96 Cell Culture Microplates. Next day, cells were treated for 24 hours with compounds. Prior to measurements, cells were incubated in DMEM assay medium in a non-$CO_2$ incubator at 37 °C for 1 h. DMEM assay medium for OCR (oxygen consumption rate) measurements contained Seahorse XF base medium (Agilent Technologies, 103335-100) including 10 mM glucose, 1 mM pyruvate and 2 mM glutamine. The following inhibitors from Seahorse XF Cell Mito Stress Test Kit (Agilent Technologies, 103015-100) were injected: oligomycin (1 μM), FCCP (1 μM), rotenone (0.5 μM), antimycin A (0.5 μM). Calculation of OCR-linked and ATP production was carried out according to instructions by the manufacturer. The DMEM assay medium for extracellular acidification rate (ECAR) measurements contained Seahorse XF base medium including 1 mM glutamine and the following compounds from Seahorse XF Glycolysis Stress Test Kit (Agilent Technologies, 103020-100) were injected: 10 mM glucose, 1 μM oligomycin A, and 50 mM 2-DG. Glycolysis rate and glycolytic capacity were calculated as described by the manufacturer by using the software Wave2.6 (Agilent Technologies). All values were normalized to the protein content of each well, measured with the DC protein assay (Bio-Rad, 5000112).

### Western blotting

MCF7 or MCF7 p53KO cells (previously described (Zhu et al., 2020)) were seeded in 6-well plates at a density of 20 × $10^4$ cells per well. Next day, cells were treated with the indicated compounds for 48 h. Cell pellets were lysed and processed as described (Popova et al., 2020). Primary antibodies used are as follows: GDF15 mouse monoclonal antibody (Sigma-Aldrich, AMAB90687), p53 mouse monoclonal antibody DO1 (Abcam, ab1101), Rabbit monoclonal antibody to ATF4 (Abcam, ab184909), Rabbit polyclonal antibody to Histone H3 (Abcam, ab1791), MDM2 mouse monoclonal antibody IF2 (Calbiochem, OP46).

### Measurement of GDF15 in cell culture medium

MCF7 or MCF7 p53KO cells were seeded in 6-well plates at a density of 20 × $10^4$ cells per well. T22-RGCΔFos-LacZ murine fibroblasts were seeded at $10^4$ cells/well in 24-well plates. Next day, cells were treated with the indicated inhibitors for 48 h and GDF15 levels in cell culture supernatant were measured using the Human (R&D Systems, DGD150) or Mouse/Rat (R&D Systems, MGD150) GDF15 Quantikine ELISA Kits.

## Experiments in db/db mice

*Experiment 1.* Seven-week old mice were randomly distributed into cages (3-4 mice per cage). Two groups were established by combining cages in order to achieve a similar weight distribution. In the first group, mice were treated daily for 27 days with 2 mg/Kg of BAY2402234 (Med Chem Express, HY-112645) administered by intraperitoneal (i.p.) injection in vehicle (5% DMSO, 40% PEG300, 5% Tween-80, 50% saline). The control group received vehicle by i.p. injection.

*Experiment 2.* Seven-week old mice were randomly distributed into 10 cages (3 mice per cage). Four groups were established by combining cages in order to achieve a similar weight distribution between groups. The first and second groups were treated with 2 mg/Kg of BAY2402234 (n=9) or its vehicle (5% DMSO, 40% PEG300, 5% Tween-80, 50 % saline) (n=9) daily via i.p. for 37 days. The third and fourth groups received 25 mg/Kg of brequinar (Bio-techne, 6196) (n=6) or saline solution (n=6) as vehicle by i.p. injection every third day until day 33. The half-life of brequinar in mice is around 12 hours according to a previous report (Sykes et al., 2016).

*Experiment 3.* 20 sixteen-week old mice were randomly distributed into 6 cages (3-4 mice per cage). Two groups of 10 mice were established by combining cages in order to achieve a similar weight distribution between groups. One group was i.p. injected every third day with 25 mg/Kg brequinar (Bio-techne, 6196) in saline solution. The control group received i.p. saline. One mouse in the saline control group showed aggressive behavior, was humanely euthanized and excluded from the study.

At the end of the experiments, mice were anaesthetized by isoflurane and euthanized by cervical dislocation. Whole blood was taken by terminal heart puncture for serum collection. Pancreases were collected and fixed as described below.

*Blood glucose and HbA1c measurements.* Blood was collected from the tail vein and blood glucose was measured using glucose strips and the glucometer from the Prima Home Test Multicare-IN meter system (BSI, 895698). Where indicated, mice were fasted for 6 hours from early morning. For measurement of glycated hemoglobin (HbA1c) Siemens DCA vantage analyzer was used.

*Intraperitoneal glucose tolerance test (IPGTT).* Mice were fasted for 6 hours from early morning. At this point, 25 mg/Kg of brequinar (Bio-techne, 6196) or saline control were administered by i.p. injection. 30 minutes later, D-glucose (Merck, G8270) was administered at 2 g/Kg by i.p. injection. Tail blood samples were taken at the indicated time-points and glucose was measured using glucose strips and the glucometer as above.

*Insulin tolerance test (ITT).* Mice were fasted for 6 hours from early morning. At this point, brequinar (Bio-techne, 6196) (25 mg/Kg) or saline solution were administered by i.p. injection. 30 minutes later, insulin (Novorapid, Novo Nordisk, A10AB05) was administered at 0.75 IU/Kg by i.p. injection. Blood was taken from the tail at the indicated times and glucose was measured as above.

*Serum measurements.* Terminal blood was collected by cardiac puncture and serum was isolated after allowing blood clotting at 4°C overnight followed by centrifugation at 1500 x g for 15 minutes. Serum GDF15 levels were measured using the Mouse/Rat GDF15 Quantikine ELISA Kit (R&D Systems, MGD150). Serum insulin was measured using a mouse insulin ELISA kit (Mercodia, 10-1247-01).

## Pancreas staining

For DAB stainings, pancreases were fixed in 4% formaldehyde for 24 h and embedded in paraffin blocks. Next, 5 μm sections were prepared, air-dried and then deparaffinized and de-hydrated using xylene and decreasing serial dilutions of ethanol. Pancreatic tissue sections were incubated for 10 minutes with 3% $H_2O_2$ in Tris-buffered saline to quench endogenous peroxidase activity. Sections were then blocked with 5% goat serum (Thermo Fisher Scientific, 50197Z) for 20 minutes at room temperature. After washing, sections were incubated with the primary antibody against insulin (1:50) diluted in blocking solution for 2 h at room temperature or overnight at 4°C. Sections were PBS-washed and incubated with secondary antibodies using IHC Select Immunoperoxidase Secondary Detection System (Millipore, DAB150) following manufacturer's instructions. Slides where counterstained with Mayer's

hematoxylin and mounted with xylene-based mounting medium. Quantifications were performed using Image J software.

For double immunofluorescence, pancreases were fixed in cold methanol and embedded in OCT freezing medium. Samples were sectioned to a mean thickness of 5 microns. After blocking with 5% goat serum, sections were then incubated overnight with anti-insulin (Santa Cruz Biotechnology, sc-8033) and anti-active caspase 3 (Cell Signaling Technology, 9664) antibody. After PBS washing, sections were incubated with Alexa Fluor 488-labeled anti-rabbit IgG (Thermo Fisher Scientific, A32731) and Alexa Fluor 555-labeled anti-mouse IgG (Thermo Fisher Scientific, A32727). Samples were mounted with mounting medium containing DAPI. Fluorescence was visualized with a Zeiss confocal laser scanning microscope (ZEN2.3 software). The fluorescence intensity of insulin, active caspase 3 and GDF15 after removal of background fluorescence was quantified by using Fiji software (ImageJ, NIH).

## QUANTIFICATION AND STATISTICAL ANALYSIS

All statistical analyses were performed using Prism software version 8.2.1 (279) (GraphPad). The tests performed are described in each figure. For all statistical tests, a p-value of $\leq 0.05$ was used to denote statistical significance (*). **, *** and **** denote $p \leq 0.01$, $p \leq 0.001$ and $\leq 0.0001$, respectively. The type of statistical test, the number of replicates and whether error bars denote standard deviation (SD) or the standard error of the mean (SEM), is specified in each figure legend.

iScience, Volume *24*

## Supplemental information

# DHODH inhibition modulates glucose metabolism and circulating GDF15, and improves metabolic balance

**Juan Zhang, Graciela Terán, Mihaela Popa, Harsha Madapura, Marcus James Graeme Watson Ladds, Danai Lianoudaki, Jacob Grünler, Marie Arsenian-Henriksson, Emmet McCormack, Martin Enrique Rottenberg, Sergiu-Bogdan Catrina, Sonia Laín, and Suhas Darekar**

## SUPPLEMENTAL FIGURES

**A**

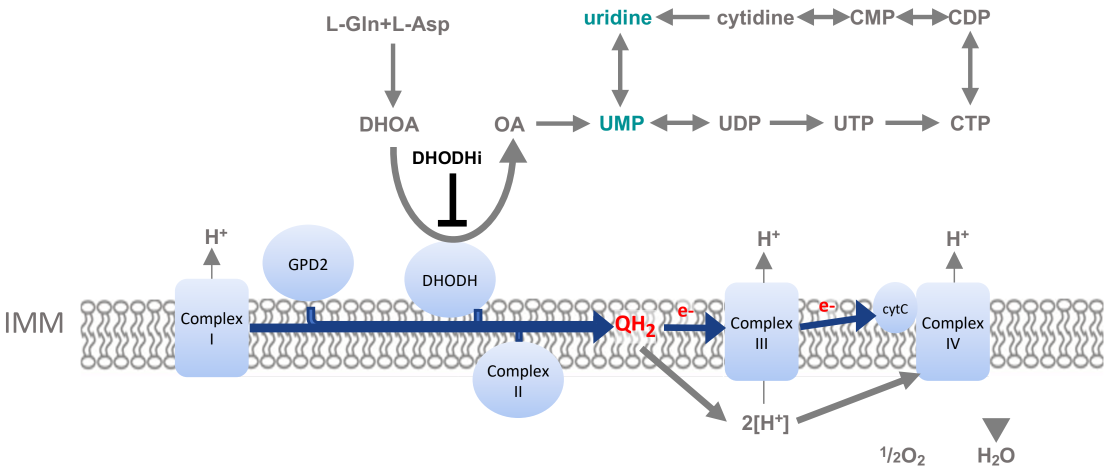


**B**

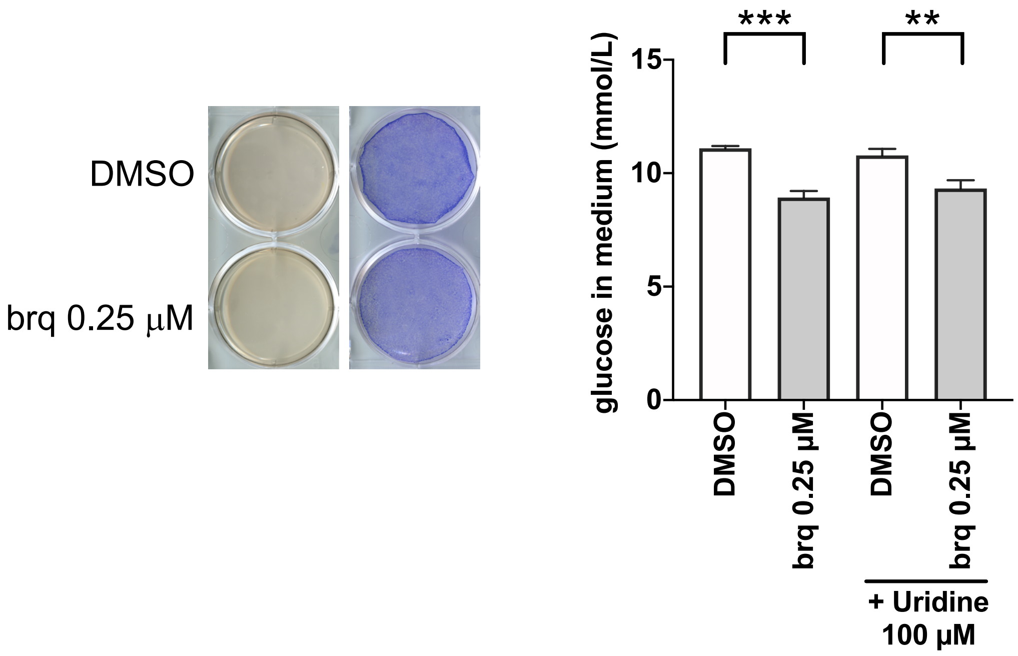


**Figure S1. (A)** Scheme depicting the function of DHODH on pyrimidine ribonucleotide synthesis and on the mitochondrial respiratory chain. IMM= inner mitochondrial membrane. Related to Figure 1.
**(B) Brequinar reduces glucose concentration in the cell culture medium.** Murine T22-RGCΔFos-LacZ fibroblasts were grown in the presence of brequinar. On day 3 after treatment, pictures of the plates were taken and Giemsa stainings were performed to assess cell density. Concentration of glucose in the medium was determined. Values correspond to the mean of three technical repeats. p values were calculated by Student's t test and error bars indicate SD. Related to Figure 1.

Figure S2

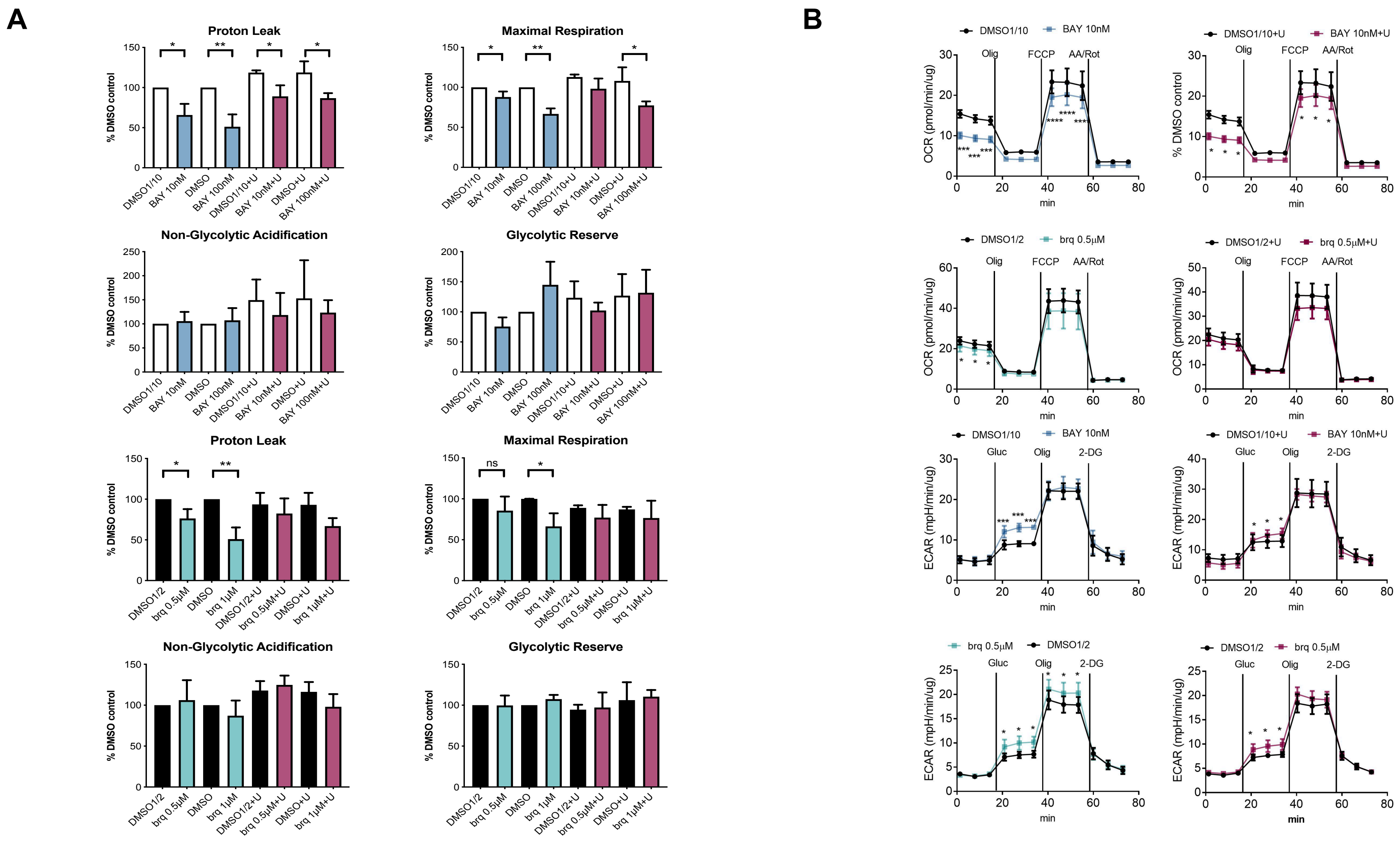


**Figure S2.** Cellular respiration and glycolysis measurements related to Figure 1C.

(A) Cellular respiration and glycolysis measurements. Values correspond to the average of 3 biological repeats and are relative to the corresponding DMSO vehicle control. Error bars represent SD and p values correspond to Student's t test. Related to Figure 1B.

(B) Average (±SEM) oxygen consumption rate (OCR) and extracellular acidification rate (ECAR) measurements. n=3 biological repeats. +U, +100 μM uridine. Related to Figure 1B.

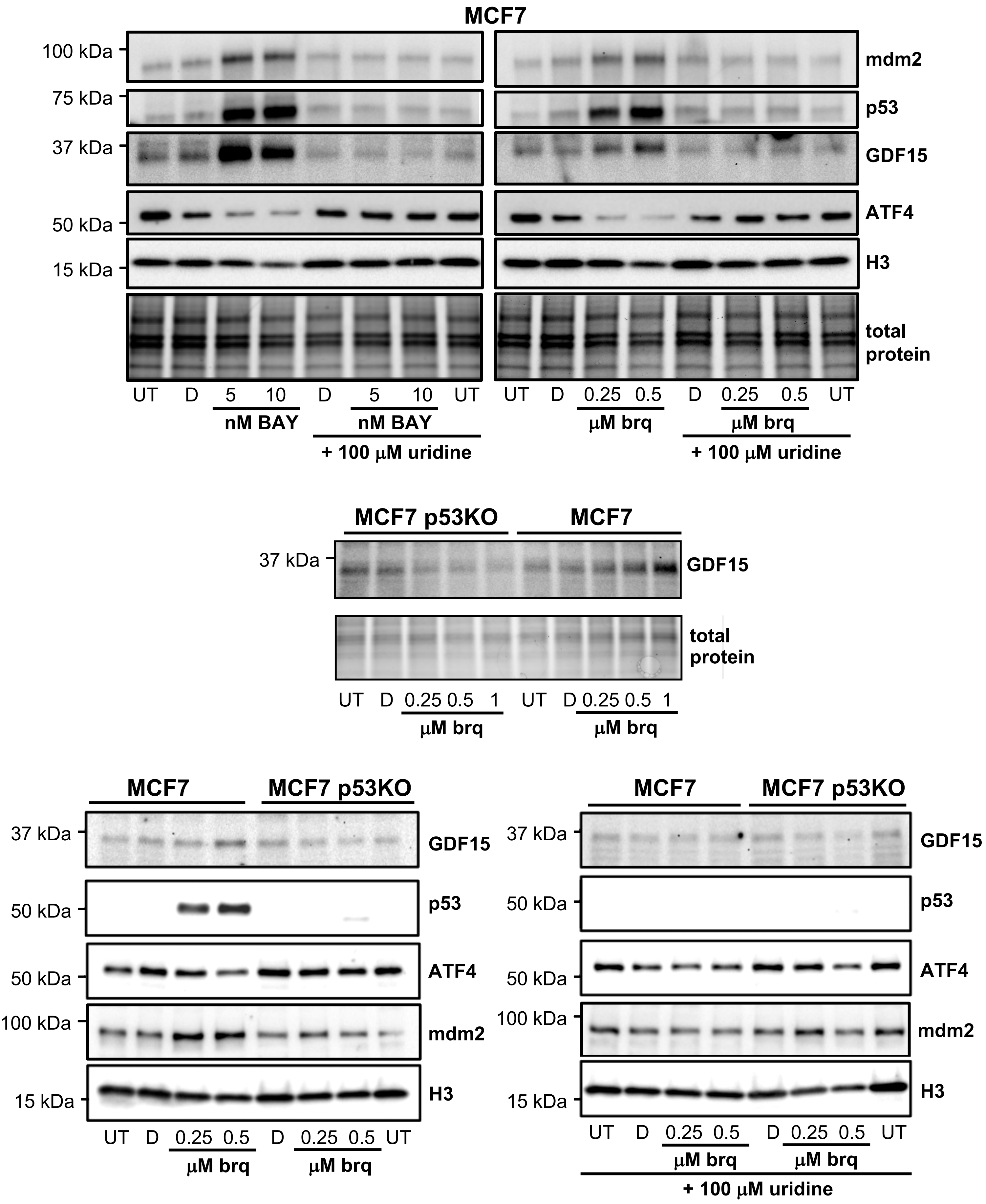


**Figure S3.** MCF7 or MCF7 p53KO cells were treated as indicated for 48 h. Expression of mdm2, p53, ATF4 and GDF15 were detected by western blotting. Total protein and histone H3 were used as loading controls. UT, untreated; D, DMSO. Related to Figure 2A.

Figure S4

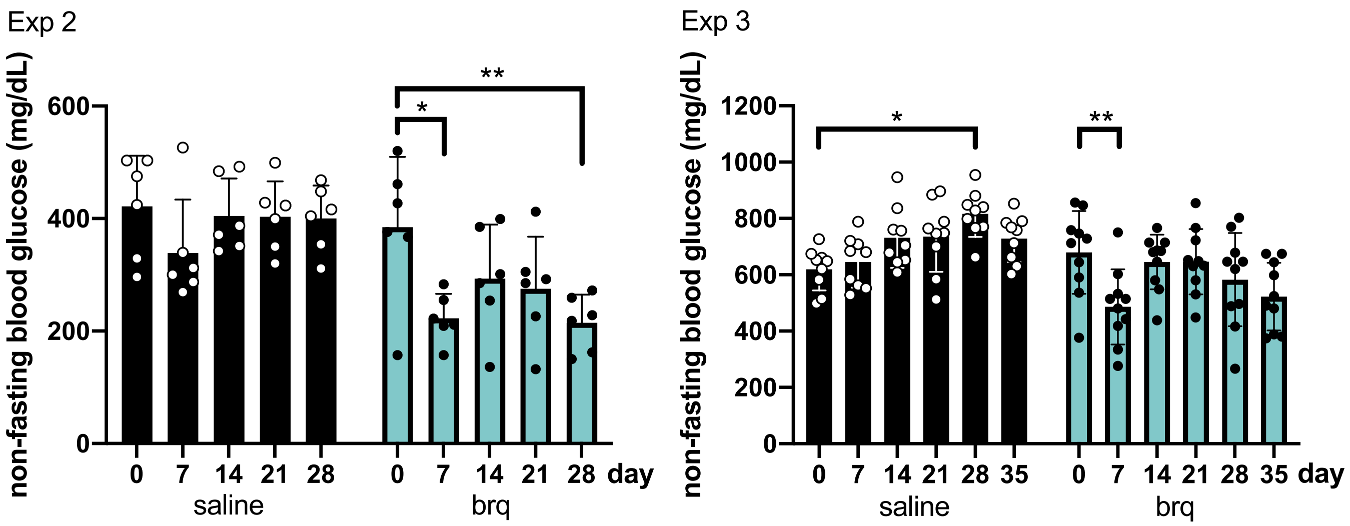


**Figure S4. Effect of DHODH inhibitors on non-fasting blood glucose.** Average (±SD) non-fasting blood glucose levels in experiments 2 and 3. Only significant changes are indicated. p values are calculated using 2way Anova. Note that in the brequinar experiments (this inhibitor is administered every three days) significance is achieved only on the days after injection (days 7 and 28). Related to Figure 5.

Figure S5

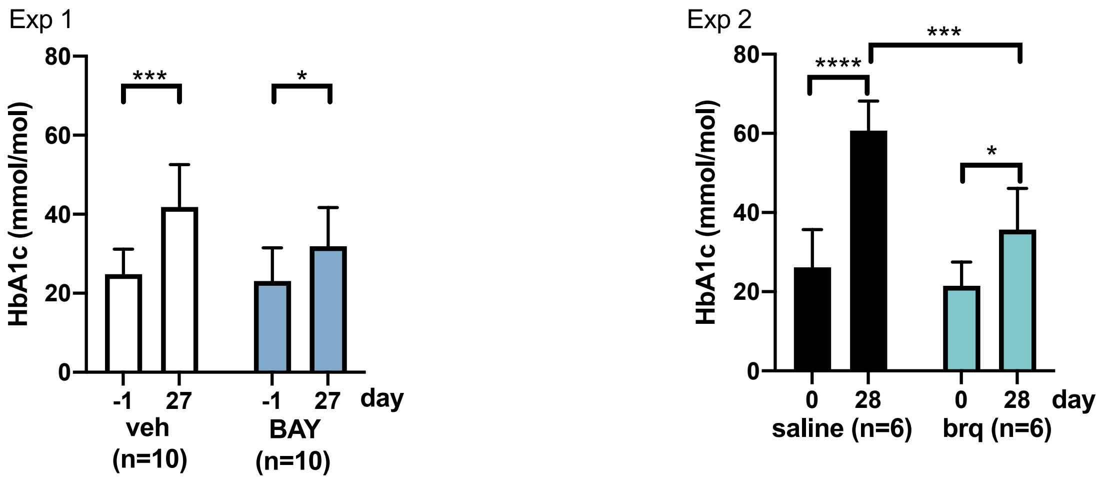


**Figure S5. Additional data for Figure 5B.** Average (±SD) HbA1c levels at the indicated days after treatment in experiments 1 and 2. p values by Student's t test. Day 0 is the treatment start day. Related to Figure 5B.

Figure S6

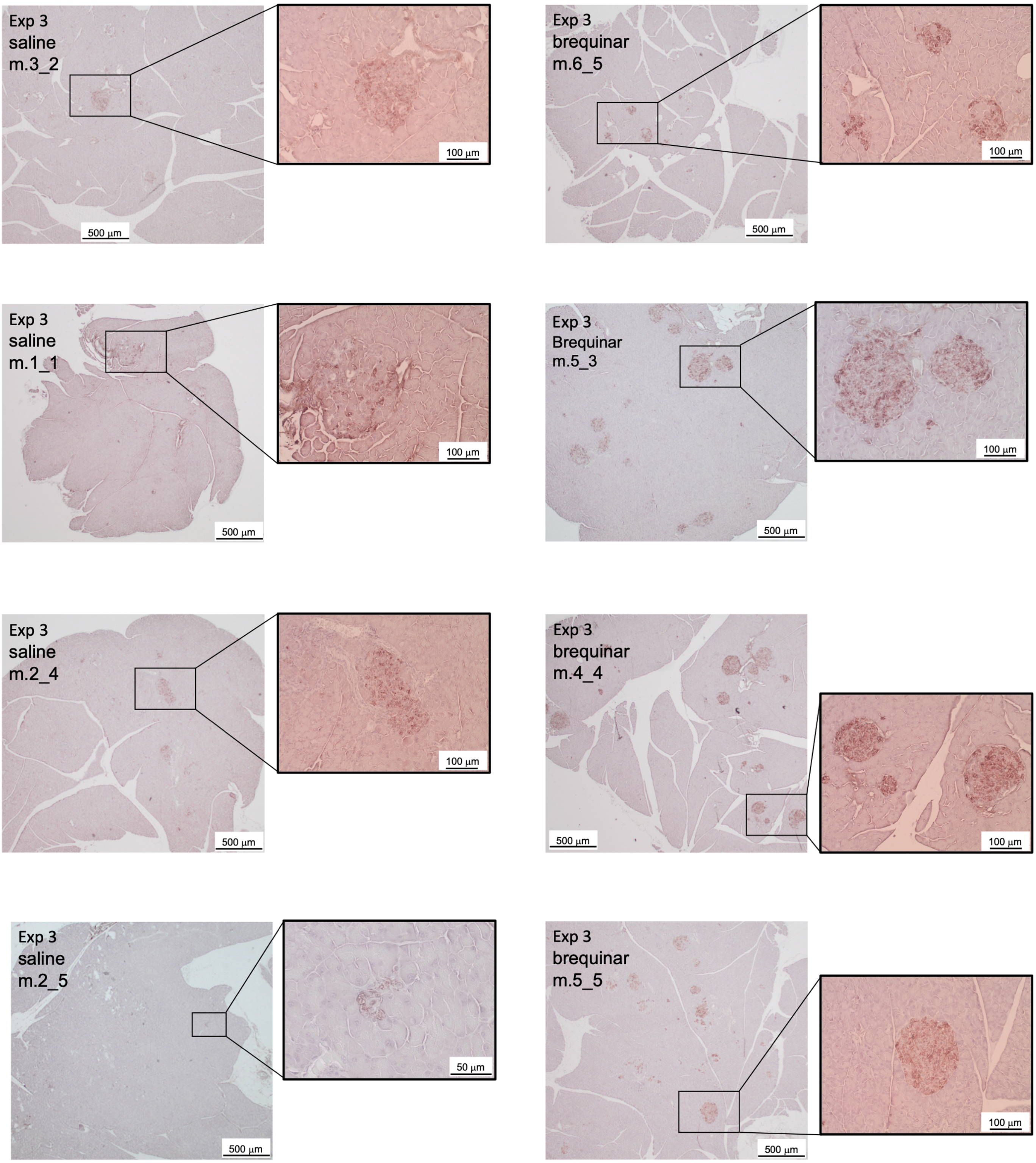


**Figure S6. Representative staining of pancreases from different mice in experiment 3 with an antibody against insulin**. Related to Figures 6B and 6C.
